# Ti6Al4V‐Bioglass‐Copper Composites for Load‐Bearing Implants

**DOI:** 10.1002/adhm.202504606

**Published:** 2026-01-26

**Authors:** Lochan Upadhayay, Bryson White, Susmita Bose, Amit Bandyopadhyay

**Affiliations:** ^1^ W. M. Keck Biomedical Materials Research Laboratory School of Mechanical and Materials Engineering Washington State University Pullman Washington USA

**Keywords:** additive manufacturing, 45S5 Bioglass, *Staphylococcus aureus*, Ti6Al4V, tribofilm, wear

## Abstract

Total hip arthroplasty (THA) and total knee arthroplasty (TKA) utilize cobalt–chromium–molybdenum alloys; however, the release of cobalt ions is a significant clinical concern. Ceramic‐based alternative systems also have concerns regarding long‐term mechanical stability. Ti6Al4V (Ti64) is a better alternative; however, it is unsuitable for articulating surfaces due to its low wear resistance. We have designed and manufactured a novel Ti64‐based composite by adding 45S5 bioglass (BG) and copper (Cu). Adding BG on titanium improves wear resistance and biocompatibility, whereas Cu addition improves mechanical strength while providing inherent lifelong bacterial resistance. Ti64, Ti64‐1 wt% BG (Ti64‐1BG), Ti64‐3 wt% BG (Ti64‐3BG), and Ti64‐3 wt.% BG‐3 wt.% (Ti64‐3BG‐3Cu) compositions were processed using the laser‐directed energy deposition (L‐DED) additive manufacturing (AM) technique. Microstructural characterisation and phase analysis were done to evaluate the influence of BG and Cu addition on Ti64. While BG was preferentially located along the melt pool boundaries, Cu was uniformly distributed throughout the sample. Uniaxial compression tests were conducted, and the addition of BG and Cu increased the strength. Biotribological analysis using flat‐on‐disc fixtures under fully immersed conditions in DMEM revealed that wear resistance improved due to the addition of BG and Cu to Ti64. Tribological testing revealed the formation of a protective nanoscale tribofilm on BG‐containing samples, as indicated by increased contact resistance and reduced wear rates at higher loads. In vitro biocompatibility studies were done with human osteoblast (OB) cells for 3 and 7 days. Cell attachment and MTT (3‐(4,5‐dimethylthiazol‐2‐yl)‐2,5‐diphenyltetrazolium bromide) assays were performed to understand the influence of BG and Cu on biocompatibility, with Ti64 serving as a control. An antibacterial test was performed for 24 and 72 h using *Staphylococcus aureus* to evaluate the influence of Cu addition on the sample's antibacterial properties. Overall, the results demonstrated a superior implant material with enhanced biocompatibility, inherent antibacterial properties, and improved wear resistance through the innovative formation of a protective nanoscale tribofilm.

## Graphical abstract

We have designed and manufactured a novel Ti64‐based composite by adding 45S5 bioglass (BG) and copper (Cu). Adding BG on titanium improves wear resistance and biocompatibility, whereas Cu addition improves mechanical strength while providing inherent lifelong bacterial resistance. Our results demonstrate a superior material with enhanced biocompatibility, inherent antibacterial properties, and improved wear resistance for load‐bearing implants.

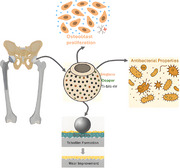

## 1 Introduction

In the US, approximately 544 000 to 790 000 hip and knee replacements are performed annually, with an expected annual growth rate of 14% [1]. Globally, hip fractures are projected to exceed 6.26 million by 2060 [2]. CoCrMo (CCM) is widely used in load‐bearing implants, particularly for hip and knee replacement devices, due to its excellent wear resistance [2]. However, when CCM femoral heads are coupled with a Ti64 stem, micromotion occurs at the interface, creating the risk of fretting corrosion along with galvanic corrosion [3]. As a result, Cobalt (Co) and Chromium (Cr) ions are released in vivo in the presence of a physiological environment [4]. Cobalt has been classified as a class 1B carcinogen material [5] by the European Commission. Adverse local tissue reaction (ALTR) is a serious lymphocyte‐mediated response to the release of metal debris in metal‐on‐metal (MoM) total hip arthroplasties (THA), which can lead to aseptic loosening, osteolysis, soft tissue destruction, pain, and morbidity [6]. For stable fixation, the tapered part of the stem should make complete contact with the tapered part of the femoral cortical bone, ensuring load transfer between the hip implants and the surrounding tissues. However, corrosion between the Ti64 stem and CCM femoral head leads to the leaching of metal ions, resulting in failure of fixation, severe pain, and the need for revision surgery. As an alternative to MoM, ceramics‐on‐ceramics (CoC) total hip arthroplasty grew popular, especially among younger patients. According to a recent case study involving 235 patients, 5 THAs required revision within 5 years, and squeaking was observed in 8 other patients [7]. Another scientific case study has reported a squeaking incidence ranging from 0.5% to 11% in THA, and squeaking hips have been associated with pain and functional impairment [8]. Initial scientific studies on CoC suggest a high failure rate, and given that millions of THAs are implanted annually, such failures can be a significant financial burden in the future. CoC may not be a viable long‐term solution. To avoid the release of cancer‐causing cobalt metal ions and minimize fretting corrosion, a better alternative to CoC is using a fully titanium‐based THA. However, poor wear resistance hinders this process. This can be achieved by creating an artificial lubricating surface between the Titanium‐based femoral head and ultrahigh molecular weight polymer liner, thus enhancing the wear resistance of titanium. To achieve this goal, titanium's wear resistance and bioactivity, including its inherent antibacterial properties, should be improved to make it suitable for use as a femoral head.

Tribofilm formation offers an alternative approach to enhancing wear resistance in various tribological systems by minimising direct contact between two surfaces. This layer typically consists of a strongly bound film that forms between two tribologically stressed surfaces, acting as a lubricant and reducing wear. Various researchers have explored different ways to improve wear properties [9]. Avila et al. enhanced the wear resistance of Ti64 by adding ceramic using AM to form an in situ Si‐based tribofilm, significantly improving hardness and reducing friction and wear [10]. Researchers have explored various surface treatments, including laser cladding, chemical vapor deposition, nitriding, and carburizing, to enhance the wear resistance of titanium‐based alloys [11, 12]. Although coating and surface treatment can improve wear resistance, delamination of the coating leads to the loss of the implants' intended mechanical properties. This work intends to create an in situ tribofilm by incorporating a soft ceramic phase, Bioglass (BG), into the Ti64 matrix.

Bioactivity refers to the ability of a material to interact with biological systems, thereby promoting a biological response at the molecular, tissue, or cellular level. Specifically, bioactive materials can interact with biological systems, creating a layer of hydroxycarbonate apatite. Bioglasses are generally an amorphous silicate material containing silica and calcium, which is known for its osteoconductive properties. They undergo dissolution, releasing silicon, calcium, and phosphate ions, which provide a conducive environment for bone growth, remineralization of acid‐damaged enamel, and bone regeneration [13]. 45S5 Bioglass is the most commonly used BG, composed of 45% SiO_2_, 24.5% CaO, 24.5% Na_2_O, and 6% P_2_O_5_ [14]. BG‐incorporated devices have received approval from the Food and Drug Administration (FDA) for clinical applications in medicine and dentistry. Mechanical and biological properties can vary based on composition, and different compositions are used in coatings, scaffolds, and small implants [15]. Moreover, ions released from bioactive glasses in vivo promote wound healing [16]. Elements such as magnesium, zinc, and copper are added to bioactive glass to enhance its biological and therapeutic functions [15]. For orthopedic implant applications, BG has been extensively studied as a coating. Although titanium is a biocompatible material, it remains inert in biological systems due to its chemical properties. Hydroxyapatite is commonly used as a coating, but BG exhibits superior osteogenic properties. Despite its good biocompatibility, standalone BG has poor mechanical properties and is brittle compared to bone, making it unsuitable for load‐bearing applications [17]. Beyond bioactiveness, BG is also known for its antibacterial properties due to its capacity to increase local pH and osmotic pressure [18].

Cu has been added to Ti64 as it is known to form a solid solution and improve strength and ductility [19, 20]. Additionally, Cu is known for its antibacterial properties. According to the Centers for Disease Control and Prevention (CDC), 35 000 die as a result of antibiotic‐resistant infection in the USA annually, and 1.27 million people die worldwide [21]. Additionally, prosthetic joint infections (PJI) account for 1.99 to 2.18% of hip replacement failures and approximately 2.05 to 2.18% of knee replacement failures, often necessitating revision surgeries to mitigate the challenges [22, 23]. Despite extensive research, the treatment failure rate for PJI is as high as 50% [24]. The current therapeutic approach involves two treatment strategies: removing infected local tissue, followed by revision surgery, and administering high doses of antibiotic drugs. However, neither of these treatment strategies is sustainable, often leading to recurring infection post‐revision surgery [23]. *Staphylococcus aureus (S. aureus)* is one of the most common microorganisms that cause infections in orthopedic devices, and it has been extensively reported to exhibit resistance to antibiotic drugs [25]. In orthopedic‐associated implant infections, biofilm formation is believed to be a key reason for antibiotic resistance, as certain antibiotics fail to penetrate the depth of the biofilm [26]. Cu has been used as an antibacterial material throughout history. The earliest documented medical application of Cu is found in one of the ancient books, the Smith Papyrus. This Egyptian manuscript, dated between 2600 and 2200 B.C, mentions using Cu for purifying and wound treatment applications [27]. It is the first antibacterial metal approved by the US Environmental Protection Agency (EPA) in 2008. Cu has been extensively studied by various researchers for its potential applications in orthopedics [27]. There have been numerous studies; however, the optimum weight percentage of Cu has been debatable [23, 28], [29]. Although 3 wt.% has no cytotoxic effect, it shows approximately 80% bacterial efficacy [23]. In this study, we added 3 wt.% Cu into Ti64 to improve mechanical properties and to provide inherent bacterial resistance.

Additive manufacturing (AM) has revolutionized manufacturing technologies by enabling the production of complex geometries with precise control over composition and shape [30]. Directed energy deposition (DED) is an advanced AM technique that operates by locally melting and fusing material layer by layer, as metal powder is delivered through a nozzle and simultaneously melted by a focused laser beam. While powder bed fusion is recommended for printing near‐net shape, laser‐DED is better suited for designing new alloys, as optimizing process parameters is easier without disturbing the print bed. Mechanical and other properties can be changed using AM, as it creates unique microstructures [31]. This study used powder‐blown L‐DED to design BG and Cu‐added Ti64 alloys.

Although BG‐coated Ti64 materials have been extensively studied, the incorporation of BG in bulk Ti64 and its effect on mechanical, microstructural, and wear properties remains poorly understood [32]. Previous research efforts on bioactive glass–titanium composites [33] lacked a systematic study and comprehensive characterization of mechanical behavior, wear properties, and biocompatibility. This work develops and characterizes additively manufactured novel titanium‐based composites reinforced with Bioglass and copper, exploring the synergetic effect on mechanical properties, wear, and biocompatibility. Compositions containing 1, 3, and 5 wt.% of BG were initially explored, but the 5 wt.% composition was excluded due to excessive brittleness. This study hypothesizes that BG will form a nano tribofilm, improving wear resistance and osseointegration. In contrast, Cu will improve mechanical strength and provide inherent antibacterial protection, making this work the first of its kind to explore the synergistic role of BG and Cu in Ti64 composites. The incorporation of BG improves wear resistance by forming a protective tribofilm. The MTT assay shows improvement in cell viability with the addition of BG. Cu reinforcement demonstrated improvement in mechanical properties, while also exhibiting intrinsic antimicrobial capabilities. This comprehensive study presents the development and characterization of new alloys, demonstrating their potential as candidates for metallic femoral head applications.

## 2 Results

### 2.1 Microstructure Evolution and Phase Analysis

**FIGURE 1:**
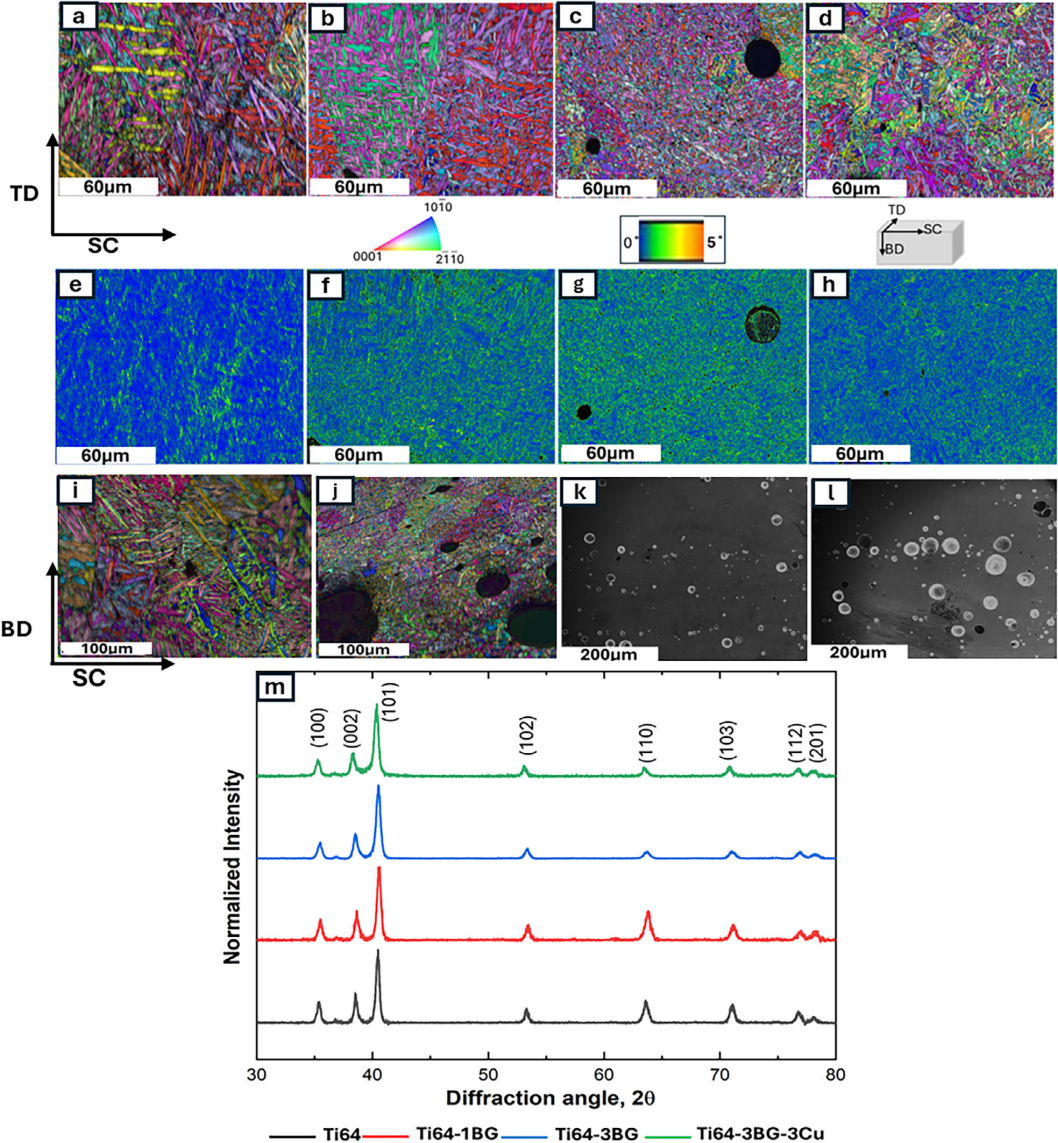
Inverse pole figure showing microstructures of DED fabricated samples showing evolution of acicular microstructures: (a) Ti64, (b) Ti64‐1BG, (c) Ti64‐3BG, (d) Ti64‐3BG‐3Cu. (e,f,g,h) shows the corresponding kernel average misorientation maps of (a), (b), (c), and (d), respectively. (i, j) show the inverse pole figure of Ti64 and Ti64‐3BG‐3Cu parallel to building directions. (k,l) shows the BG particles distributed along the edge of the melt pool for Ti64‐1Bg and Ti64‐3Bg compositions. All samples exhibit acicular microstructures. The black regions correspond to unindexed regions containing BG particles during the EBSD scan. (m) XRD patterns for all sample compositions, confirming the presence of the α/α' phase of Ti64.

EBSD microstructures of DED‐manufactured samples are shown in Figure 1a–d along the longitudinal section perpendicular to the building directions. EBSD data with a confidence index (CI) less than 0.1 µm were removed for analysis. Standardized cleanup with a minimum grain tolerance angle of 2 degrees was performed. Other than standard cleanup, no other data manipulation was performed. For kernel average misorientation (KAM), only CI < 0.1 filtering was done, and the first nearest neighbors were used for measurement. Acicular α' is the predominant microstructure for control and treatment samples. These microstructures result from high cooling rates during DED, which are 10^3^–10^4^ K/s. Cooling rate higher than 410°C/S leads to the formation of a complete martensitic transformation from β to α' phase [34]. For such rapid cooling, the temperature of the underlying material must be lower than the martensite transformation temperature. Most of the β phase transforms to the α' phase via a shear martensitic phase transformation [35]. Figure 1e–h shows the kernel average misorientation map for all compositions. Misorientation was higher for all treatments compared to the control due to the addition of BG and Cu. A higher amount of BG leads to an increase in KAM value. These increased misorientations could lead to strain localization around alpha grain boundaries, promoting crack initiation. The area average was used to quantify lath microstructures, utilizing ferret min and max diameters, as these matrices better represent non‐spherical or elongated shaped microstructures. Maximum ferret diameter decreased from 14 µm to 6 µm with the addition of 3 wt. % BG and Cu, whereas ferret min diameter decreases from 4.3 to 2.8 µm. Decrement in acicular alpha needle length is higher compared to that of width. Lath width was measured using the number average to compare with the published literature. BG acts as a heterogeneous nucleation site, which is likely the reason for reducing the alpha lath length by adding BG. Cu is a beta stabilizer, refines prior beta grains as well as lath microstructures; similar results have also been reported [36]. Figure 1i,j shows the IPF map of Ti64 and Ti64‐3BG‐3Cu along the build direction. Columnar grains are found in both samples along the build direction. The Ti64‐3Bg‐3Cu sample shows refined prior beta columnar grains. Interestingly, refined grains were observed near the unindexed regions, indicating BG serves as a grain refiner. BG particles are located along the melt pool, as shown in Figure 1k,l for 1BG and 3Cu samples, because they have a lower density and are positioned around the edges of the melt pool. The BG melting temperature is lower than that of Ti64; irregular‐shaped BG particles change to spherical‐shaped particles, as evident in Figure 1k. Particle size varies from about 7 to 70 µm with a mean size of 30 µm. Particle distribution (Figure S1) was similar for all treatment compositions. The particle size for the BG‐added sample was smaller due to particle breakage during ball milling. The quantitative analysis of alpha lath length and thickness is presented in Table 1, showing a progressive reduction in alpha lath length and thickness with the addition of BG. The lath is further refined due to the addition of Cu, exhibiting a synergistic effect on microstructural refinement.

**TABLE 1:** Alpha lath dimensions for all compositions.

Compositions	Max ferret diameter (µm)	Min ferret diameter area average (µm)	Min ferret diameter number average (µm)
Ti64	14.34 ± 8.4	4.34 ± 3.4	1.2 ± 1.0
Ti64‐1BG	12.08 ± 8.7	3.6 ± 3.02	1.09 ± 1.0
Ti64‐3BG	6.7 ± 5.8	3.2 ±2.5	0.84 ± 0.7
Ti64‐3BG‐3Cu	6 ± 4.2	2.8 ± 2	0.75 ± 0.6

XRD patterns of Ti64, Ti64‐1BG, Ti64‐3BG, and Ti64‐3BG‐3Cu samples are shown in Figure 1m, which indicates the presence of α/α'. Both α and α' have hexagonal structures; hence, it is challenging to differentiate between α and α'. No amount of β was observed from the XRD analysis, as it might be present in small quantities and difficult to detect using XRD. Additionally, the beta phase was not detected through EBSD. The addition of BG and Cu to Ti64 did not favor the formation of the β phase during fabrication using L‐DED. Since BG exists in an amorphous (glassy) phase, it does not exhibit any peak in the XRD pattern.

### 2.2 Mechanical Properties

**FIGURE 2:**
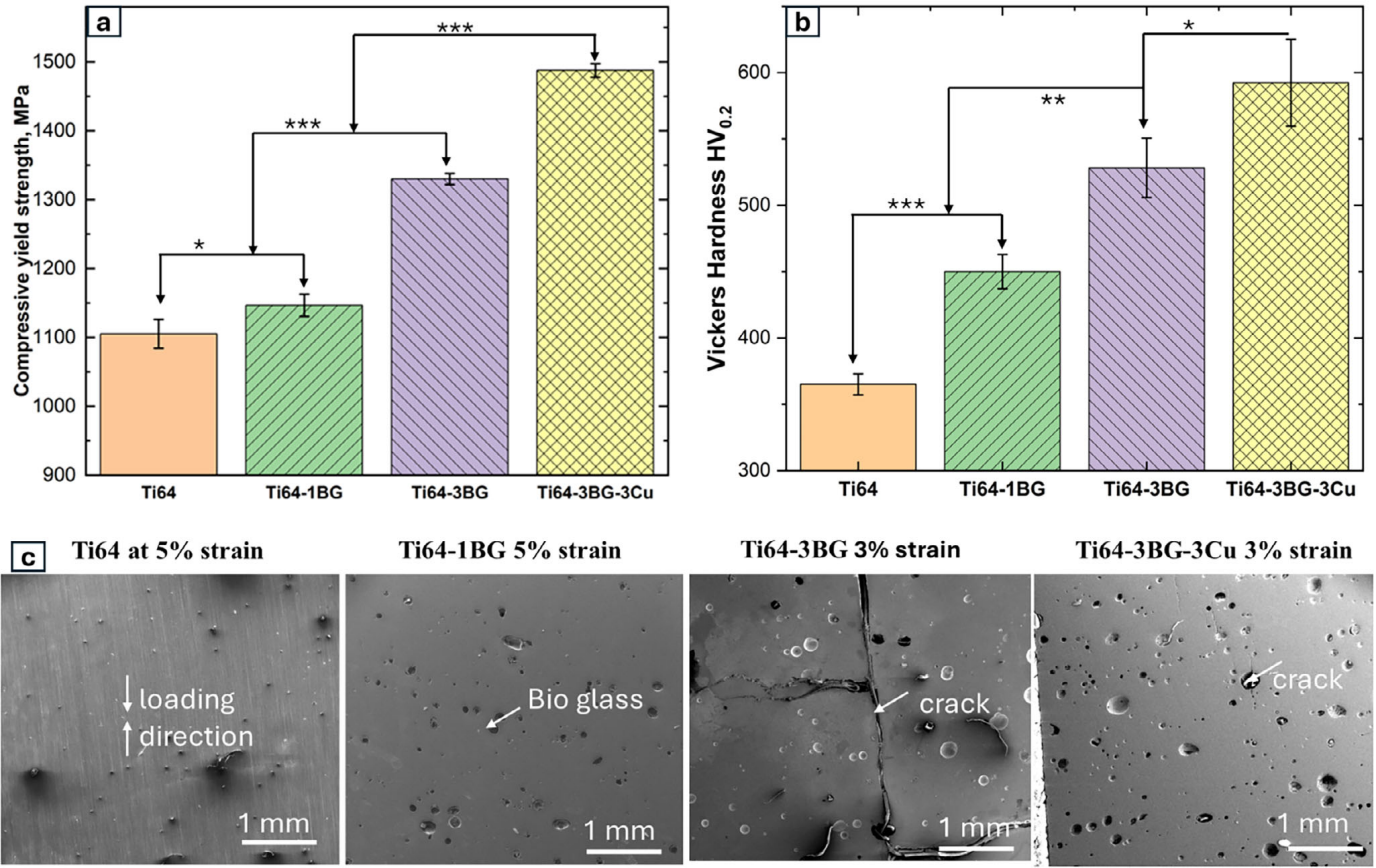
(a) Plot showing the compressive yield strength of all compositions (n=3). (b) Graphical representation of the Vickers hardness values of each sample (*n* = 2). (c) SEM micrographs of the sample post‐compression test, Ti64‐3BG, and the Ti64‐3BG‐3Cu sample showing crack formation. All data represented shows the mean ± standard deviation.

The compressive yield strength was calculated using the 0.2% offset method. Compressive yield strength for Ti64, Ti64‐1BG, Ti64‐3BG, and Ti64‐3BG‐3Cu is shown with a bar graph in Figure 2a. Ti64 shows a compressive yield strength of about 1100 MPa, and the addition of 3 wt.% of BG increased it to 1330 MPa. Alloying 3 wt. % of BG and 3 wt.% of Cu improved the compressive yield strength of Ti64 from 1100 to 1490 MPa. Vickers hardness exhibits similar trends in mechanical properties as observed in compression tests. The Ti64 sample exhibited the lowest hardness value, with 365 ± 9 HV_0.2_. Adding BG and Cu showed improvement in hardness: 450 ± 12 for Ti64‐1BG, 528 ± 22 HV_0.2_ for Ti64‐3BG, and a maximum hardness of 592 ± 32 HV_0.2_ for Ti64‐3BG‐3Cu. This represents a significant hardness improvement of up to 62% compared to the base Ti64 alloy. The increase in variation of hardness can be attributed to inhomogenous distribution of BG particles. The hardness‐to‐yield strength ratio increases from 3.23 for Ti64 to 3.90 for Ti64‐3BG‐3Cu samples. An increase in the hardness‐to‐yield strength ratio suggests increased resistance to deformation, at the expense of ductility. No significant change in modulus was found upon adding copper and Bioglass as evident from nano‐indentation tests (shown in Figure S2). Following compression testing, samples were sectioned and examined to assess the deformation and failure mechanisms of the materials. Sectioning was necessary as standard compression tests provide limited insight regarding the ductility behavior of materials. SEM micrographs of compression tested for all samples are shown in Figure 2c. No cracks were observed in the Ti64 and Ti64‐1BG samples after the post‐compression test at 5% strain. In contrast, large cracks were seen in Ti64‐3BG samples after 3% strain. These cracks were located at random locations originating from the BG particle due to strain mismatch. Ti64‐3BG‐3Cu shows improved ductility compared to the Ti64‐3BG sample, as evident from the 3Cu post‐compression sample. Interestingly, small cracks originating from BG particles were seen in Ti64‐3BG‐3Cu, though ductility is inferior compared to that of Ti64 and Ti64‐1BG samples. Overall, while strength increases with the addition of BG, ductility decreases. Adding Cu, however, improves both strength and ductility.

### 2.3 Tribological Test

**FIGURE 3:**
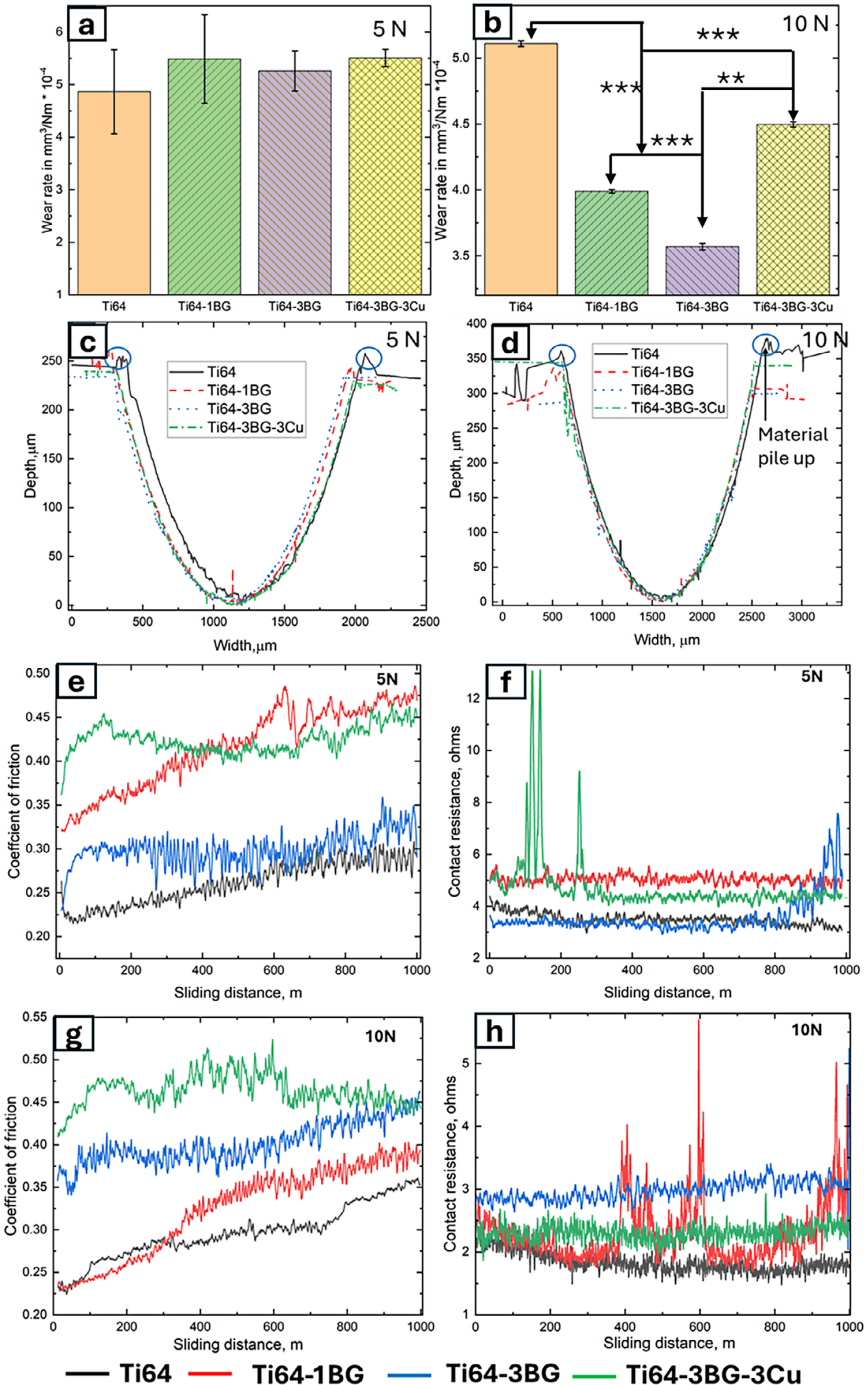
(a,b) Normalized wear rates for 5 and 10N, respectively. (c,d) Plot showing representative line scans along the cross‐section of the wear scar for 5 and 10 N, respectively (n=3). (e, g) Evolution of the coefficient of friction with sliding distance at 5 and 10 N loading, respectively. (f, h) Variation of contact resistance with sliding distance at 5 and 10 N indicates the formation of a protective tribofilm at higher loads. All tests were conducted in DMEM at room temperature with a reciprocating distance of 1000 m and a 1 Hz frequency. All data are represented as mean ± standard deviations. *** represents *p* <0.001, ** represents *p* <0.01, * represents *p* <0.05.

Normalized wear rates were accurately measured and presented in Figure 3a,b under loading of both 5 and 10 N. At a lower load of 5 N, no statistically significant difference in wear rate was observed across all compositions. However, when a 10 N load was applied, a notable improvement in wear rate was observed with the addition of 1 and 3 wt.% BG. This suggests that tribological performance is load‐dependent, with elevated Hertzian contact stress, BG shows improved wear resistance. The wear rate of Ti64 for 5 N is 4.86 ± 0.8 mm^3^/Nm; all other composition wear rates fall within the error margin of the Ti64 sample. On the other hand, wear rate for Ti64, Ti64‐1BG, Ti64‐3BG, and Ti64‐3BG‐3Cu are 5.1 ±0.02, 3.98 ± 0.01, 3.56 ±0.02 and 4.45 ± 0.02 mm^3^/Nm, respectively. The wear rate for all treatment compositions is lower than that of the control Ti64 at a 10 N load. The variations in wear rate decrease as the applied load increases from 5 to 10 N for all treatments. However, the normalized wear rate for the base alloy (Ti64) remained unchanged when the load was increased. All the wear tracks displayed a U‐shaped cross‐section wear profile. Interestingly, material pile‐up behavior was observed in Ti64 and 1BG samples along the edge of the cross‐section parallel to recipropcating direction, as highlighted by the circle, indicating the ductility behavior of the materials. In contrast, clean edges were observed for Ti64‐3BG and Ti64‐3Cu samples. Line profiles along the cross‐sectional areas perpendicular to reciprocating motion directions are shown in Figure 3c,d for 5 and 10 N, respectively. In addition to the wear rate, the width and depth of the wear scar are calculated from the line profile shown in Figure 3c,d and tabulated in Table 2. Wear and scar measurements align with the wear rate, as a 5 N load does not show the difference in wear scar width and depth. With a 10 N load, the width and scar of wear tracks decreased for BG and Cu addition compared to the control Ti64 sample. Among all compositions, Ti64 showed the widest and deepest wear track, consistent with the wear rate for 10 N. Wear scar width and depth are the smallest for Ti64‐3BG. For 5 N, wear scar depth varies from 241 to 271 µm, and wear scar width varies from 1622 to 1708 µm across all compositions, whereas for 10 N, wear scar depth and width vary from 293 to 269 to 1812 to 2087 µm. Wear scar depth and width increased by 50% and 26%, respectively, for Ti64, whereas they increased by 21% and 17% for the Ti64‐3BG sample as the load increased from 5 to 10 N. Across all compositions, the increase in wear scar depth exhibits a higher percentage increase than the increase in wear scar width.

**TABLE 2:** Presents the wear scar width and depth for loads of 5 and 10N, as calculated from the optical profilometer measurements.

Load	Compositions	Wear scar depth,µm	Wear scar width,µm
5N	Ti64	247 ± 27	1662 ± 50
	Ti64‐1BG	257 ± 18	1703 ± 61
	Ti64‐3BG	241 ± 20	1622 ± 56
	Ti64‐3BG‐3Cu	271 ± 46	1708 ± 37
10N	Ti64	369 ± 9	2087 ± 81
	Ti64‐1BG	323 ± 13	1812 ± 41
	Ti64‐3BG	293 ± 11	1907 ±32
	Ti64‐3BG‐3Cu	341 ± 7	2040 ± 41

To further study tribological behaviour and the formation of the tribofilm layer, the coefficient of friction (COF) and contact resistance (CR) were measured during the tests and are shown in Figure 3. For both 5 and 10 N loading conditions (Figure 3e,g), treatment compositions show a higher COF value than the control (Ti64). COF for Ti64‐3BG and Ti64‐3BG‐3Cu varies from 0.35 to 0.5 N for both loads, whereas for Ti64 and Ti64‐1BG, COF varies from 0.22 to 0.35. In general, higher COF represents more resistance to sliding, often leading to strong interfacial adhesion or the formation of a protective film, which increases friction but prevents further wear. Interestingly, Ti64‐3BG and Ti64‐3BG‐3Cu exhibit steady‐state attainment wear behavior for both 5 and 10 N loads. In contrast, the COF for Ti64 and Ti64‐1BG continues to increase as the test progresses, suggesting that 1 wt.% is insufficient to alter the wear mechanisms. BG particles are embedded in the titanium matrix, which are likely to debond and be released during initial wear. Notably, the initial fluctuation on COF is attributed to abrasive interaction and the microploughing effect caused by BG particles. However, beyond 150 m, COF begins to stabilize at 5 N for Ti64‐3BG and Ti64‐3BG‐3Cu samples (see Figure 3e), indicating a transition to a steady‐state wear regime under sliding conditions. Under a high applied load of 10 N, the initial fluctuation reduces and transitions to a steady‐state wear regime within 100 m for the Ti64‐3BG and Ti64‐3BG‐3Cu samples (Figure 3e,g). These results suggest that higher normal loads facilitate faster promotion of a stable surface. Contact resistance was measured throughout the test by passing a small current through the counter ball and maintaining continuity to the samples. Contact resistance has been used to study the formation of in situ tribofilm in titanium‐based alloys [37]. As shown in Figure 3f,h, contact resistance reveals insight into interfacial stability. A distinctive pattern in contact resistance is observed as the load increases from 5 to 10 N. Contact resistance varies from 4 to 6 ohms for a 5 N load, and from 2 to 5.5 Ω while testing under 10 N. Notably, for both loading conditions, all BG‐reinforced composite shows higher contact resistance values. Intermittent spikes in contact resistance are possibly due to the accumulation of debris or the formation of a transient tribofilm layer. Moreover, higher fluctuation was observed during testing at 10 N, suggesting a local breakdown or formation of a tribolayer. A notable phenomenon was observed in the evolution of contact resistance as the test progressed. As the test progresses, the wear ball also wears out, and the initial point contact changes to an area contact. Also, resistance is indirectly proportional to area; theoretically, contact resistance decreases as the test progresses. This expected contact resistance was observed in the Ti64 sample while loading under 5 and 10 N. In contrast to this behavior, BG‐reinforced treatment compositions exhibit an incremental evolution of contact resistance. This peculiar behaviour suggests the formation of a tribofilm, which increases the contact resistance. Similar results have been reported when reinforcing ceramic particles on Ti64 [37].

**FIGURE 4:**
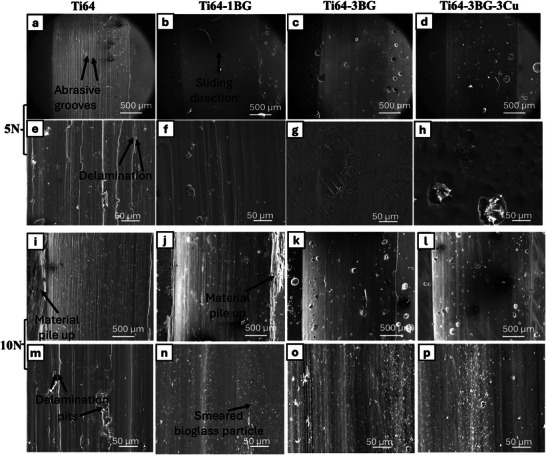
SEM micrographs of all compositions after 5 N (a–h) and 10 N (i–p) after wear tests, evidencing the wear track becoming smoother with BG addition. Smeared BG was found along the wear track after the 10 N loading condition.

Figure 4 shows SEM micrographs of the wear track after post‐testing under applied normal loads of 5 and 10 N. These micrographs provide critical insights into wear mechanisms. Abrasive wear occurs when hard particles or asperities cut or plow other surfaces. Ti64 samples exhibit pronounced abrasive wear behavior with both 5 and 10 N loading conditions, characterized by large abrasive grooves along the wear surface. These features indicate the cutting and ploughing action of hard particles during tribotesting. Significant formation of surface pits and large groove wear was observed in the wear track (shown in Figure 4a,e,i,j). In contrast, Ti64‐3BG and Ti64‐3BG‐3Cu samples did not show the presence of any grooves or layer delamination. With an increment in BG wt. % Wear track surface smoothness increases. Pits formed in Ti64‐3BG and Ti64‐3BG‐3Cu samples due to BG particles being debinded during wear. Often, these holes are pits that become filled with BG particles due to wear, as the BG particles become smeared. On Ti64‐3BG and Ti64‐3BG‐3Cu samples, a thick oxide layer was formed, as evidenced by the high‐magnification image shown in Figure 4g,h. The Ti64 and Ti64‐1BG samples exhibit significant plastic deformation, with material accumulation along the edges (as shown in Figure 4i,j) under a 10 N load. A higher load exhibits more pronounced delamination and subsurface cracking, resulting in greater plastic deformation. Upon 10 N loading, BG particles become smeared and fragmented, forming particulates along the wear track, as seen in Figure 4n–p. These smeared particles vary in size from nanometers to a few micrometers. EDS was performed on large particles, with a few micrometre‐sized particles shown in the supplementary section (Figure S3). Due to EDS's spatial resolution limitations, smaller particles could not be effectively identified using EDS. Tribological testing demonstrated the formation of a protective nanoscale tribofilm on BG‐containing samples, as evidenced by increased contact resistance and reduced wear rates at higher loads. This was observed for Ti64‐1BG, Ti64‐3BG, and Ti64‐3BG‐3Cu, respectively, after 36 h.

### 2.4 Cytocompatibility Analysis

**FIGURE 5:**
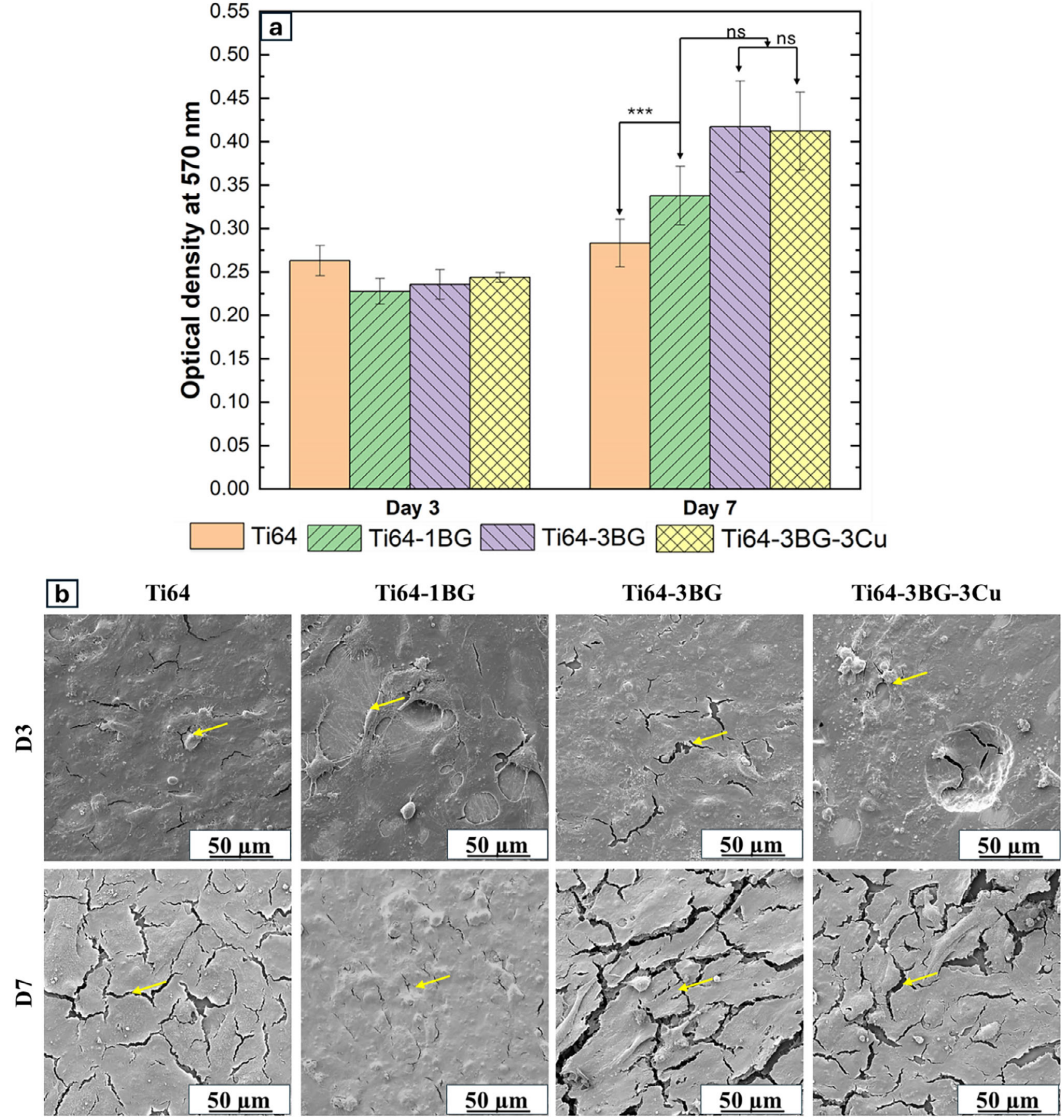
(a) MTT assay results after 3 and 7 days demonstrating that all compositions exhibit excellent cytocompatibility with osteoblast cells (All data are represented as mean ± standard deviations, *** denotes *p* < 0.001, ns is not significant, n = 3). (b) SEM micrographs after 3 and 7 days reveal healthy cell morphology on all substrates, confirming good cell attachment and proliferation across all compositions. Yellow arrows point to healthy osteoblast cells.

MTT assay results in Figure 5a demonstrate enhanced osteoblast cell viability in BG and Cu‐modified Ti64 composites. After 7 days, Ti64‐1BG, Ti64‐3BG, and Ti64‐3BG‐3Cu exhibited 19%, 47%, and 46% higher cell viability, respectively, compared to the control Ti64. These results indicate that the modified composites exhibit superior cytocompatibility. SEM images in Figure 5b further support these findings, showing healthy, well‐spread osteoblast cell morphologies on all substrates at both day 3 and day 7. The cells maintained a flattened, adherent morphology, indicating robust attachment and proliferation. No signs of cytotoxicity or abnormal cell behavior were observed in any of the samples. These results collectively confirm that the addition of BG and Cu to Ti64 significantly enhances cell viability and supports healthy osteoblast growth, demonstrating excellent cytocompatibility.

### 2.5 In Vitro Bacterial Viability Test

**FIGURE 6:**
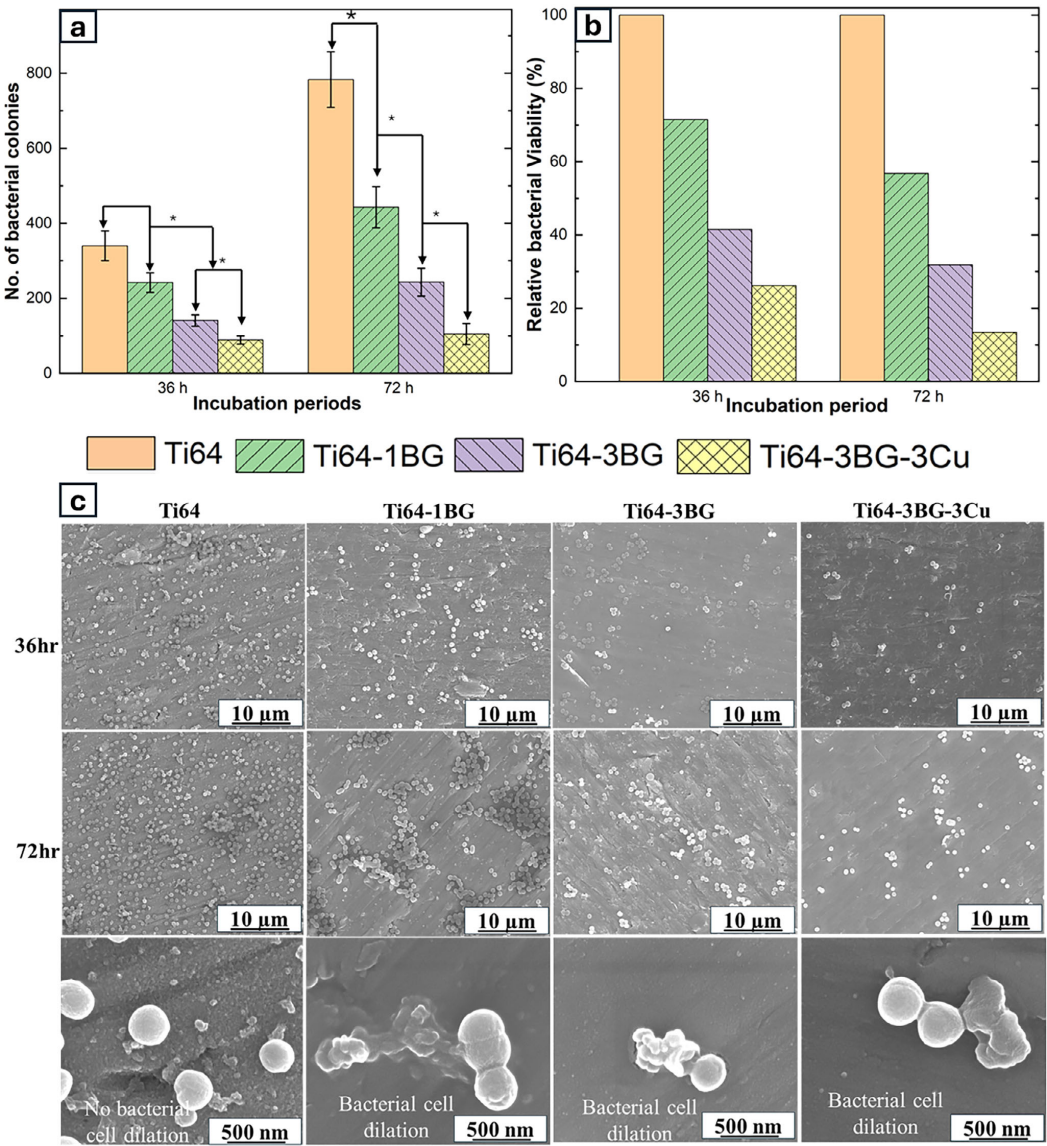
(a) Graphical representation of the number of *S. aureus* CFU after 36 and 72 h for all compositions. (b) Plot showing relative bacterial cell viability compared to the control after 36 h and 72 h. (c) SEM micrographs of bacterial colonies after 36 and 72 h, showing increased bacterial colonies on Ti64 samples, whereas BG and Cu showed a comparatively reduced bacterial population and revealed dilated cell morphologies. High magnification images show bacterial cell dilation on Ti64‐1BG, Ti64‐3BG, and Ti64‐3BG‐3Cu samples. All data are represented as mean ± standard deviations,*** represents *p* <0.001, ** represents *p* <0.01, * represents *p* <0.05, n=3.SEM image displaying S. aureus bacteria morphology for 36 and 72 h on the metal substrate of all compositions.

Bacterial infection at the implant site remains a prevalent complication in orthopedic applications. Ti64 does not have inherent antibacterial properties. At the same time, Cu and BG have been recognized as antibacterial materials that resist bacterial growth. As shown in Figure 6a, the bacterial colonies decreased from 340 for the control Ti64 to 240, 141, and 89. This represents a progressive improvement in antibacterial properties with increasing BG content. Cu shows a synergistic effect on antibacterial efficacy with the addition of BG. The antibacterial mechanism of BG has been attributed to two main mechanisms: pH increases due to the dissolution of BG and an increase in osmotic pressure resulting from the release of BG ions, such as Na^+^ and Ca^2+^ [38]. Numerous studies have demonstrated that copper (Cu) exerts its antibacterial properties by generating reactive oxygen species (ROS), resulting in oxidative stress and damage to cellular structures [39, 40, 41]. The control sample showed bacterial growth, increasing from 340 to 783 colonies after 72 h of bacterial incubation whereas, the BG and Cu‐reinforced samples maintained low bacterial proliferation. This sustained inherent antibacterial activity suggests that BG and Cu can inhibit bacterial growth and colonization for orthopedic devices. Figure 6b illustrates the relative bacterial viability of the treatment sample compared to the control. The addition of 1 wt% and 3 wt% reduced the bacterial percentage by 70% and 50%, respectively, after 36 h, and further reduced it to about 60% and 30%, respectively, after 72 h. The most significant antibacterial effect was observed in Ti64‐3BG‐3Cu, which reduced bacterial viability by about 75% and 85% after 36 h and 72 h, respectively.

Figure 6c shows the morphology of *S. aureus* bacteria after an in vitro viability test. Notably, with the addition of BG, the bacterial count decreased, demonstrating the antibacterial effect of Cu. Moreover, the bacterial count continued to decrease with the addition of Cu. Ti64 showed an increase in bacterial count for 72 h compared to 36 h. High‐magnification images of the bacterial sample on a Ti64 substrate show healthy bacterial morphology. In contrast, bacterial cell dilation with distorted morphology was seen in treatment samples. SEM micrographs qualitatively support the antibacterial effect of BG and Cu.

## 3 Discussions

### 3.1 Effect of BG on Mechanical and Microstructural Properties

Ti64 alloys consist of aluminum and vanadium, which are alpha and beta stabilizers, respectively. Both alpha and beta phases are present in conventional cast alloys, with their shape and size depending on heat treatment and thermomechanical processing. However, the microstructural features of the AM‐manufactured samples are complex and governed by the cooling rates, which may not be comparable to the cast product [42]. During the DED process, Ti64 powder undergoes melting, transforming to the β phase, and upon cooling, transforms back to the α phase. Crystallographic orientation between BCC beta titanium and HCP alpha titanium is well established, which is represented by a Burgers orientation relationship and given by {110}_α_ || {0001}_β_ and <1–11>_α_ || <‐1120>_β_. For a given burger relationship, there could be 12 crystallographic variants. Those variants can be represented by 5 distinct α, β transformation types [43]. Our disorientation angle suggests strong burger orientation relationships. The boundary angle at 60 corresponds to <11–20>, which is the dominant boundary, accounting for approximately 50% of the total boundaries. In contrast, peaks at 10, 63, and 90 degrees represent other types of α/α boundaries. Parent grain reconstruction was performed using TSL OIM 9.0 following the Burger orientation relationship (shown in Figure S4). Prior Beta grain size decreased from 66 µm to 27 µm when 3 wt.% BG was added to Ti64. The size and thickness of β grains depend on the thermal cycle and the periods between β transus temperature and the melting temperature [42]. Previous studies on AM of Ti64 have reported beta grain thicknesses ranging from 103 µm to 4 mm [44]. Prior β grains are generally larger for the DED sample than for LPBF and EBM [44, 45].

The α phase preferentially nucleates at the prior β grain boundaries. All compositions in our study predominantly displayed basket‐wave microstructures, which are the most common and widely reported [44, 46]. DED cooling can vary from 10^3^–10^5o^K/S, leading to the formation of the α' martensite phase. Cooling rate exceeding 410°K/S leads to the formation of the complete α’phase [34]. Our XRD results confirm the formation of α/α' without any detectable β phase. Cooling rates for SLM and DED are somewhat similar, although the melt pool for DED is way larger than that of SLM. Hence, basket‐wave with α' dominant microstructures are expected in the DED and PBF Ti64 sample. Previous studies have reported alpha lath dimensions in DED Ti64 ranging from 0.6–0.7 µm, as reported in [45], which aligns with our observations of 0.7 µm.

Ceramic particles have been reported to refine prior β in titanium‐based alloys during solidification [47]. For instance, reinforcement with TiB ceramic particles has been shown to reduce lath thickness from 1.4 ± 0.12 to 0.68 ± 0.1 µm with a 5% TiB addition through laser melting deposition, attributed to TiB serving as nucleation sites [47]. Similarly, in our study, the BG particle acts as a heterogeneous nucleation site, reducing the alpha lath length and thickness from 18 to 4.48 µm and from 0.7 to 0.4 µm, respectively. The spheroidization of BG particles observed in Figure 1k indicates that BG melts but maintains its compositional integrity, allowing it to function as an effective nucleation agent during resolidification. The thermal conductivity difference between Ti64 (approximately 6.7 W/mK) and BG (approximately 0.9–1.1 W/mK) induces local thermal gradients along the melt pool, altering solidification kinetics. The local thermal variation occurs when the BG gets melted, further refining the lath microstructures. While some studies have reported only the α' phase presence [48], others have demonstrated varying proportions of α and β phases by modifying dwell time during hatching [49].

The martensitic α' is a non‐equilibrium phase characterized by distorted lattice structures, which induce lattice strain, making it stronger than the conventional α+β microstructure. Additionally, a high dislocation density is induced by martensitic formation, which enhances the material's strength through dislocation strengthening. Reducing alpha lath thickness and prior beta grain boundary improves the strength of Ti64 due to grain boundary strengthening as predicted by Hall–Petch theory. Generally, AM‐produced Ti64 exhibits higher strength compared to wrought Ti64, albeit at the expense of ductility. Grain boundary strengthening is a key factor contributing to the notable increase in yield strength, enhancing it from 1100 MPa in Ti64 to 1490 MPa in the Ti64‐3BG‐3Cu alloy. Lin et al. reported that adding 1.2 wt.% predicted determined Thermo‐Calc calculations into Ti64 using wire arc directed energy deposition (DED) increased the yield strength from 868 MPa to 934 MPa [19]. The study also observed a reduction in both β grains and α‐phase size, with no formation of intermetallic compounds. In our study, no formation of Cu–titanium was observed in the XRD analysis, and a uniform distribution of Cu was observed via EDS (Figure S3). Both solid solution and grain boundary strengthening contribute to the strengthening effect of Cu in Ti64 [19].

Beyond grain boundary strengthening, the undissolved BG particles, as shown in Figure 1e,f, impede dislocations and bow dislocations around the particles, requiring higher stress to overcome them, improving yield strength. A study by He et al. demonstrated dislocation pileup around La_2_0_3_ (ceramic particle) on LaB_6_/Ti64 composites, attributing the phenomenon to strength improvement [50]. Studies on adding TiC‐TiB2 nanoparticles have shown similar results with dislocation pile‐up around nanoparticles [51]. With the addition of LaB_6_ and TiC‐TiB_2_ on Ti64, yield strength improved by about 14.5% and 17%, and increased by about 25% with the addition of 3 wt. % BG in Ti64. As anticipated, the trade‐off between strength and ductility was observed prominently in the Ti64‐3BG sample. The elastic modulus of Ti64 is 110 GPa, whereas the modulus of fully dense BG 45S5 is 78 GPa [52]. This mismatch in modulus causes stress concentration at the BG particle matrix interface during loading, leading to crack initiation, Figure 2c. The improvement in ductility observed with Cu addition has been attributed to the reduced aspect ratio (length to thickness) of alpha laths [53]. Additional studies have attributed this phenomenon to the refinement of both α lath and prior β grains, emphasizing Cu's role as a β stabilizer. During AM thermal cycling, this causes residual β to transform into soft, ductile α lath, thereby enhancing overall ductility.

### 3.2 Wear Mechanisms

Adding BG and Cu improved wear resistance, particularly at higher loads (10 N), indicating a load‐dependent tribological mechanism. At a lower load of 5 N, no statistically significant difference in wear rate was found, suggesting that a minimal threshold contact stress is required to improve wear rate. COF measures the ratio of the friction force to the normal load. COF variation is similar for both 5 and 10 N, indicating the contribution of adhesive and abrasive wear is comparable in both cases. Although a smooth surface in BG with added titanium was observed (Figure 4), this indicates an adhesive‐controlled wear mechanism. We can conclude that the wear mechanism is not dominated by adhesive and abrasive wear behaviour for 5 N. A higher contact resistance for both 5 and 10 N has been observed when BG is added, providing evidence of tribofilm formation. Contact resistance measurement has been previously utilized to confirm tribofilm formation [10, 29, 54]. Tribofilm is a nanoscale mechanism, and real‐time monitoring of tribofilm formation has been studied by [55], using Nano Infrared spectroscopy and time‐of‐flight secondary mass ion spectroscopy. A previous study identified a 50–150 nm thick zinc‐dialkyldithiophosphate tribofilm that was studied on a steel surface using atomic force microscopy [56]. The study also reported that the tribofilm formation mechanism was time‐ and stress‐dependent. Although hardness increased significantly, no direct relationship was observed between wear rate and hardness, as predicted by Archard's equation, further indicating that multiple mechanisms contribute to improved wear resistance. The addition of BG and Cu resists plastic deformation, as evident from the wear track micrograph and the optical profilometer line scan (Figure 3c,d).

**FIGURE 7:**
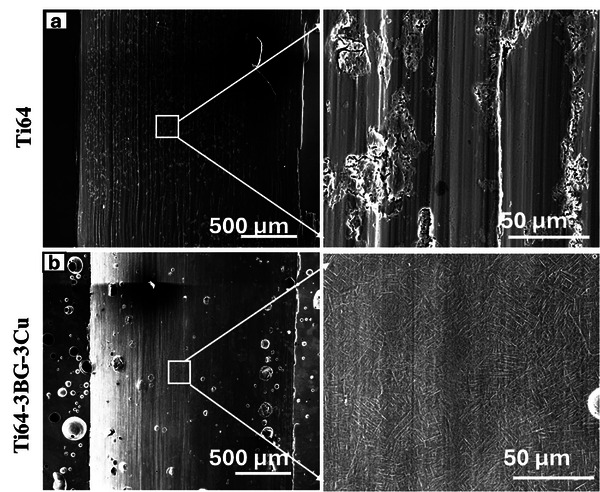
SEM micrographs of lightly worn track, (a) Ti64, (b) Ti64‐3BG. Localized attacks were observed in the Ti64 wear track, whereas acicular microstructures were observed in the Ti64‐3BG wear track.

Fine, smeared BG particles were found along the wear track of the BG‐added Ti64 sample, forming a tribofilm that acts as a solid lubricant, thereby reducing metal‐to‐metal contact. This tribofilm comprises mixed oxides of the wearing materials, such as BG particles. The formed tribofilm is electrically insulating, which increases contact resistance. Contact resistance increases as the test progresses, although it is expected to decrease as the contact area increases, providing another indirect indication of tribofilm formation. The gradual increase in contact resistance as testing progressed (Figure 3b,d), despite the expected decrease as the contact area increases, is another indirect indication of progressive tribofilm formation, consistent with findings reported in previous studies [54, 57]. To further verify tribofilm formation, wear track samples were lightly etched for 10 s using the Keller reagent, as described in the Materials and Methods section. Etched wear track micrographs for Ti64 and Ti64‐3BG are shown in Figure 7. Typical additive‐manufactured acicular microstructures were observed along the track of the Ti64‐3BG sample, Figure 7b. Upon etching, the tribofilm gets completely removed, showing further evidence of tribofilm formation. Small pits are observed in Ti64‐3BG wear tracks due to the removal of BG particles from the wear track during etching. Similar results were observed for Ti64‐1BG and Ti64‐3BG‐3Cu, which are presented in Figure S5. In contrast, a Ti64 exhibits localized corrosion attack rather than revealing acicular microstructures. This is due to pits, deep wear scratches, and a lack of tribofilm, causing localized damage. No direct relationship was found between Lath size and its effect on wear rate.

### 3.3 Cell Viability and Antibacterial Activity

Ideally, implants should be able to support cell attachment and proliferation. BG is known for its ability to form a hydroxycarbonate apatite (HCA) layer, which enhances both osteogenesis and angiogenesis, promoting bone growth while withstanding physiological stress [58]. This HCA layer mimics the mineral phase on natural bone, providing a favourable surface for osteoblast cell attachment, proliferation, and differentiation. Our results show an increase in MTT value (Figure 5), indicating improved cell viability for BG‐added samples.

Adding BG and Cu to Ti64 demonstrated enhanced antibacterial properties compared to the base alloy, which is critical for orthopedic and dental implants. The antibacterial properties of BG have been hypothesized to release alkali and alkaline earth metals, which elevate the pH and increase local osmotic pressure by disrupting the cell membrane [18, 59]. The resulting alkaline environment makes the environment stressful for bacteria, causing them to alter shape and internal structures by adjusting gene levels and proteins [60]. A 98% in bactericidal efficacy was reported with 45S5 BG powder utilizing a particle size of 50 µm at a concentration of 50 mg/ml [18]. In a separate study, to achieve a 6‐log reduction in *S. aureus* biofilm, the concentration had to be increased to 500 mg/mL using a 45 µm particle diameter, despite a slight difference in methodology [59]. Another study reported an 8‐log reduction of *S. aureus* biofilm using 50 µm with a concentration of 50 mg/mL [61]. Bioactive glass antibacterial efficacy is dose‐dependent, as indicated by a study conducted in 16 species using 100 mg/mL of S53P4 [62].

Material surface chemistry is one of the primary factors influencing bacterial colonization. The bactericidal efficacy of Cu depends on various factors, including but not limited to the physical form of Cu, Cu oxidation state, proximity of bacteria to Cu‐containing surface, and environmental conditions [23]. In literature, the exact mechanism of Cu bactericidal action remains uncertain; however, contact killing is the most widely accepted mechanism [39]. We performed a release study of all control and treatment samples, along with commercially pure Cu with the same exposed area, using X‐ray fluroscence (XRF) microscopy to verify this. All control and treatment samples show no release of Cu ions, as their concentration is similar to that of the blank solution, suggesting that the Cu ions originate from the digesting HNO_3_ media. However, commercial Cu shows 200 times higher ionic release of Cu with a concentration of about 740 ppm (shown in the supplementary section Figure S5). Our XRF measurement suggests that the actual killing mechanism of Cu for 3 days is contact killing, and Cu doesn't get released in 3 days as it forms a solid solution. No Ti ion release was also observed, as indicated by XRF. The bacterial killing process involves the disruption of the bactericidal membrane due to Cu^2+,^ followed by damage to the membrane, leakage of cytoplasmic contents, generation of reactive oxygen species (ROS), and finally, DNA damage [39]. Several studies have reported that the antibacterial properties of Ti64, when alloyed with Cu, have been fabricated using various manufacturing methods [28, 63, 64]. However, there is a lack of consensus on the exact sequence of events, as different testing protocols, variations in strains, and experimental conditions can significantly impact the results [65]. Investigation into membrane damage mechanisms has revealed nonenzymatic oxidative degradation of phospholipid layers, which plays a crucial role in compromising membrane integrity, leading to bacterial death on different Cu surfaces. This process was found to have efficacy of about 70–99.9% against the E. coli bacterial strain [65]. Other key contributors to Cu's bacterial killing are due to the generation of oxidative stress. Upon membrane damage, Cu ions can penetrate the bacterial membrane, increasing the concentration of Cu ions in various oxidation states, including Cu, Cu(I), and Cu(II) [41]. Cu produces hydroxyl ions under aerobic conditions, damaging lipids, proteins, and nucleic acids by releasing ROS [66, 67]. In Summary, Cu's antimicrobial activity is well established; however, the precise mechanism is still debatable. With the addition of BG and Cu, our material exhibits synergistically improved inherent antibacterial properties, making it a promising candidate for biomedical applications.

## 4 Conclusion

This study demonstrated the development of BG and Cu‐added Ti64 composites using laser‐directed energy deposition (L‐DED), offering a promising solution to address the inherent limitations of conventional Ti64 implants, particularly for articulating surfaces. Our comprehensive studies into microstructural, mechanical, tribological, biological, and antibacterial properties have yielded several significant findings.

1. Adding BG and Cu to the Ti64 refined microstructure, with BG acting as a nucleation site, most notably reduced the α Lath length from 14 µm to 6 µm and the thickness reduction from 1.2 µm to 0.75 µm in the Ti64‐3BG‐3Cu compositions. The acicular α' martensitic structure remained predominant across all compositions, as a characteristic microstructure of AM'ed Ti64.
2. Mechanical properties were enhanced with the addition of BG and Cu. Compressive yield strength increased from 1100 MPa in Ti64 to 1490 MPa in Ti64‐3BG‐3Cu, representing a 35% improvement. Similarly, hardness values improved by up to 62%, reaching 592 HV in Ti64‐3BG‐3Cu compared to 365 HV in the base alloy. Such increments were attributed to grain boundary strengthening, reduction in lath morphology, and dispersion strengthening due to the addition of BG. While adding 3 wt.% BG reduced ductility, including Cu, helped mitigate this effect, providing a better balance between strength and ductility in the Ti64‐3BG‐3Cu composition.
3. Tribological performance demonstrated load‐dependent behavior, with significant improvements in wear resistance observed at higher loads (10 N) due to tribofilm formation. The wear rate decreased by approximately 30% in Ti64‐3BG compared to Ti64 under 10 N loading. The formation of a protective tribofilm on BG‐containing alloys was confirmed through contact resistance measurements and post‐wear microscopic analysis.
4. Biocompatibility assessments via MTT assays confirmed that BG addition enhanced osteoblast cell viability and attachment, addressing the bio‐inertness limitation of conventional Ti64. Cu addition did not exhibit any cytotoxic effect on cell viability.
5. Incorporating BG and Cu imparted inherent antibacterial properties to the Ti64 alloy. Bacterial viability was reduced by approximately 75% and 85% in Ti64‐3BG‐3Cu after 36 and 72 h, respectively, compared to the control Ti64 sample. This sustained antibacterial efficacy demonstrates the potential for these alloys to provide long‐term protection against infection without relying on antibiotic administration.

The synergistic effects of combining BG and Cu in Ti64 create a multifunctional titanium BG metal glass composite system that simultaneously addresses multiple critical requirements for load‐bearing implants, enhancing wear resistance through tribofilm formation, improving mechanical properties, improving biocompatibility, and offering inherent bacterial resistance. Such results offer a significant advancement in alloy design for biomedical applications using AM to reduce implant failure rates and improve patient outcomes.

## 5 Materials and Methods

### 5.1 Materials Design and Fabrication

In this study, commercial powder was used for the manufacturing process. Ti64 (TEKMAT), BG (MatExcel), and Cu (Carpenter). All powders were sieved before use. The following compositions were prepared. Ti64 was used as the control material. Ti64 was modified by adding one weight percent of BG Ti64‐1 wt.% BG (Ti64‐1BG), and three weight percent of BG, Ti64‐3 wt.% BG (Ti64‐3BG). BG addition was limited to 3 wt. %, as higher wt. % increased brittleness and was difficult to machine. 3 wt.% of BG and 3 wt.% of Cu (Ti64‐3BG‐3Cu) were added to study the effect of Cu in the BG‐added titanium composite. Powder was premixed and ball milled using a mechanical rolling machine at 50 rpm for 5 h with zirconia (ZrO_2_) milling media (one‐third by powder weight). All samples were fabricated using powder‐blown Laser‐DED (Formalloy), equipped with a 1‐kilowatt fiber laser. All powder was fed through a hopper using a powder‐blown L‐DED delivery system and printed in an argon environment, with oxygen levels below 100 ppm. Two sample geometries were fabricated: rectangular samples of 15 x 17 x 5.1 mm were printed in a CP‐Ti base plate for microstructure and tribological studies, and cylindrical samples were printed for in vitro cell and bacterial studies, as well as for compression testing (Shown in Figure S1g). Process parameters were optimized by varying laser power and scanning speed through numerous trials to get the desired geometry. The final optimized parameters are presented in Table 3. Layer thickness and hatch spacing were kept constant at 0.3 mm and 0.8 mm, respectively. Infill, hatch power, and speed were kept the same, and the powder disc rate was maintained at 0.3 rpm. Volmetric energy density was calculated as:

(1) $$\mathrm{Volumetric}\;\mathrm{energy}\;\mathrm{density}\;\left(J/mm^{3}\right)=\frac{P}{V\;\times h\times t\;}$$

where p is the power in watts, v is the speed in mm/s, h is the hatch spacing in mm, and t is the thickness in mm. Post printing, all samples underwent stress‐relieving heat treatment at 450°C for 2 h in a vacuum of approximately 0.02 MPa using an Across International tubular furnace.

**TABLE 3:** Optimised process parameters for various compositions using L‐DED manufactured.

Compositions	Infill power (W)	Speed (mm/min)	Gas flow rate (liters/min)	Energy density (J/mm^3^)
Ti64	300	1100	9	68.21
Ti64‐1BG	300	950	9	78.94
Ti64‐3BG	280	780	13	89.74
Ti64‐3BG‐3Cu	280	820	13	85.36

### 5.2 Microstructure and Phase Analysis

The top section of all samples was prepared for microstructural analysis by grinding upto 1200‐grit silicon carbide paper, followed by polishing with alumina to 0.01 µm using an automatic grinding and polishing machine (5N load per sample, 120 rpm). Grinding was performed for 120 s for each grade, while polishing was performed for 300 s at each step. Mirror‐finished samples were sonicated in ethanol and etched in Kroll's reagent (2 mL of HF, 4 mL of HNO_3_, and 50 mL of DI water) for 15 s. A post‐etching microstructure was observed using an EDS‐equipped scanning electron microscope (FESEM, FEI, OR, USA). X‐ray diffraction (XRD) was performed to study the phase using Cu‐Kα radiation, with a wavelength of 1.541 Å, at 40 kV voltage and 15 mA current. Scanning was performed from 30 to 80 degrees with a step size of 0.1 Å. Elemental distribution was evaluated using SEM energy dispersive spectroscopy (SEM‐EDS). Samples for electron backscattered diffraction (EBSD) were ground and polished to 0.05 µm using alumina suspension solution, followed by vibromet polishing in 0.05 µm colloidal silica particles for 20 h. Samples were thoroughly cleaned with soapy water and immediately subjected to EBSD Scanning. EBSD was performed on the top surface of the sample along the scanning direction (SD) and the transverse direction (TD) using 20 kV with a step size of 0.15 µm using the EDAX Velocity pro EBSD system. Additionally, EBSD scans were conducted along the build direction (BD), approximately 0.5 µm from the top surface of the build plane, with a step size of 0.4 µm. Data analysis was performed using TSL OIM 9.0 software.

### 5.3 Mechanical Testing

Samples were machined into cylindrical specimens to a 5 mm diameter and 9 mm height using a milling machine. The height‐to‐diameter ratio was maintained between 1.5 and 2, as per the ASTM E9‐19 standard, to avoid bulging and buckling effects. Three samples were tested for each composition. Cylindricity was verified before testing. Compression tests were performed using an Instron 600 DX universal testing machine with a strain rate of 10^−3^/S at room temperature. A 500 N pre‐load was applied before starting the test, with the load applied parallel to the build direction in compression testing. Vickers' hardness measurements were performed on the top surface of all compositions after grinding with P1200 SiC grinding paper. Using a phase II plus Vickers hardness testing machine, a 200 gf load was applied with a dwell time of 15 s, with a spacing of atleast 500 µm between two indents.

### 5.4 Bio‐Tribocorrosion Testing

Bio‐tribocorrosion testing was conducted using a linear reciprocating ball on a flat test setup, utilising a DUCOM biotribometer in accordance with the ASTM G133 standard. All samples were mounted, ground, and polished to the same roughness, with testing conducted only on the top surface to ensure consistency, with 3 samples tested for each composition. The backside of the sample was grounded to make contact with the Cu electrode for contact resistance measurement, with electrical continuity ensured throughout the test. The ball was securely fastened in the holder to prevent rotation during testing. A 3 mm Tungsten carbide wear ball was used for the testing. Wear Test was performed in biologically relevant Dulbecco's Modified Eagle Medium (DMEM) (Sigma–Aldrich), with a sliding distance of 1000 m, linear sliding speed of 0.02 m/s. Tests were performed at both 5 and 10 N loads to study the effect of load on wear behavior. The coefficient of friction was determined by measuring the horizontal and normal load throughout the test. Post tribological testing, wear track measurement was performed using a Zygo optical profilometer. The obtained data were processed using Gwydion software to accurately measure the cross‐sectional area of the wear track by integrating the area under the curves. The exact wear rate of material was calculated for all compositions as follows:

(2) $$Normalized\;Wear\;rate=\frac{wear\;volume\;\left(mm^{3}\right)}{sliding\;distance\;\left(m\right)*load\left(N\right)\;}$$

Wear tracks were also observed under SEM, and EDS was performed to study elemental compositions along the wear track.

### 5.5 In Vitro Cytocompatibility Testing

The cytocompatibility of all substrate compositions toward human fetal osteoblasts (HFOB, American Type Culture Collection (ATCC)) cells was determined using previously reported methods [67]. The cells were cultured in a growth medium comprised of DI water, Dulbecco's Modified Eagle's Medium (DMEM, Sigma–Aldrich), G418 (Sigma–Aldrich), NaHCO_3_ (Sigma–Aldrich), 10% fetal bovine serum (FBS, ATCC), and 1% penicillin/streptomycin (ATCC). The cells were incubated at 37°C with 5% CO_2_ in this medium until at least 80% confluency was achieved. Cell seeding was performed following previously reported methods to load each sample with 25000 cells. Cells were allowed to attach for 30 min in an incubator before being added to the cell media. After 3 and 7 days of incubation, the viability of osteoblast cells was assessed through an MTT [3‐(4,5‐dimethylthiazol‐2‐yl)‐2,5‐diphenyltetrazolium bromide] assay. This was performed by aspirating the media from each sample's well, then transferring the samples to a new well plate before adding 300 µL of MTT reagent along wth 1.7 mL of cell media. These samples were then incubated for 4 h before the media was aspirated, and 600 µL of an MTT solubilizer solution, comprising isopropanol (Avantor), Triton X‐100 (Sigma–Aldrich), and 0.1 M HCl (Sigma–Aldrich), was added dropwise. 100 µL aliquots were transferred to a 96‐well plate, and the optical density was read using a UV–Vis spectrophotometer (Biotek Synergy 2) at 570 nm. SEM imaging was employed after 3 and 7 days of incubation to analyze cell morphologies. For this, samples were fixed with a solution containing 2% paraformaldehyde and 2% glutaraldehyde in a 0.1 M phosphate buffer for 24 h. Then, the samples were washed with 0.1 M PBS and treated with 2% osmium tetroxide for 1 h, followed by serial dehydration in hydroethanolic solutions and drying with hexamethyldisilazane (HMDS). Samples were gold sputter‐coated (Jeol, DII‐29010SCTR Smart Coater) and imaged.

### 5.6 In Vitro Bacterial Testing

In vitro bactericidal properties were evaluated for Ti64, Ti64‐1BG, Ti64‐3BG, and Ti64‐3BG‐3Cu according to ISO 22196:2011 guidelines, using previously reported procedures [67]. Bactericidal properties were evaluated against the control (Ti64), the current standard material used in various orthopedic and dental applications. *S. aureus*, a Gram‐positive bacterial strain (Carolina Biological, Burlington, NC, USA), was used to evaluate the bacterial response of all treatments, including the control. Using the McFarland scale, the *S. aureus* bacterial strain in freeze‐dried conditions was rehydrated and diluted to achieve an optical density of 1.5 × 10^8^ colony‐forming units (CFU). Before testing, the sample was ground and sterilized in an autoclave at 120°C for an hour. Sterilized samples were placed in a 24‐well plate, and 10^6^ CFU of bacteria were loaded in triplicate samples and allowed to attach for an hour, followed by adding 1 mL of nutrient broth. Samples were incubated at 37°C for predetermined times of 36 h and 72 h, respectively. After predetermined intervals, the nutrient broth was removed and fixed with a fixative (2% paraformaldehyde and 2% glutaraldehyde, respectively) in 0.1 M PBS and kept overnight. After cleaning with PBS three times, 2% osmium tetroxide was added to the sample for 3 h at ambient temperature. Progressive dehydration was performed using a graduated ethanol serial dilution from 30% to 100%. These ethanol‐treated samples were then treated with hexamethyldisilane (HMDS) for critical point drying. A gold coating was applied before SEM imaging. The number of bacterial colonies was counted using the DotDotGoose open‐source software. Antibacterial efficacy was calculated using the following:

(3) $$Antibacterialefficacy=\frac{N_{control}-N_{treatment}}{N_{control}}\times 100$$

wheras *N_control_* is the number of bacterial colonies in the control sample (Ti64) and *N_treatment_* is the number of samples in the treatment compositions.

### 5.7 Statistical Analysis

All results are expressed as mean ± standard deviation. Statistical comparisons were performed using one‐way analysis of variance, followed by Tukey's test for multiple group comparisons. All statistical analysis was performed using Origin Pro. *p* < 0.05, *p *< 0.01 and *p *< 0.001 represents *, **, *** respectively for statistical significance.

## Conflicts of Interest

The authors declare no conflicts of interest.

## Supporting information




**Supporting File 1**: adhm70775‐sup‐0001‐SuppMat.docx.

**Supporting File 2**: adhm70775‐sup‐0002‐3cu KAM ‐ Fig 1h.jpg.

**Supporting File 3**: adhm70775‐sup‐0003‐Ti64 KAM ‐ Fig 1e.jpg.

## Data Availability

The data that support the findings of this study are available on request from the corresponding author. The data are not publicly available due to privacy or ethical restrictions.
