# Supplementary material for: Ti6Al4V‐Bioglass‐Copper Composites for Load‐Bearing Implants
Source: Adv Healthc Mater. 2026 Jan 26;15(13):e04606. doi: 10.1002/adhm.202504606 (PMC13058786; doi:10.1002/adhm.202504606)
Supplement: Supplementary file 1 — Supporting File 1: adhm70775‐sup‐0001‐SuppMat.docx. [file ADHM-15-0-s003.docx]

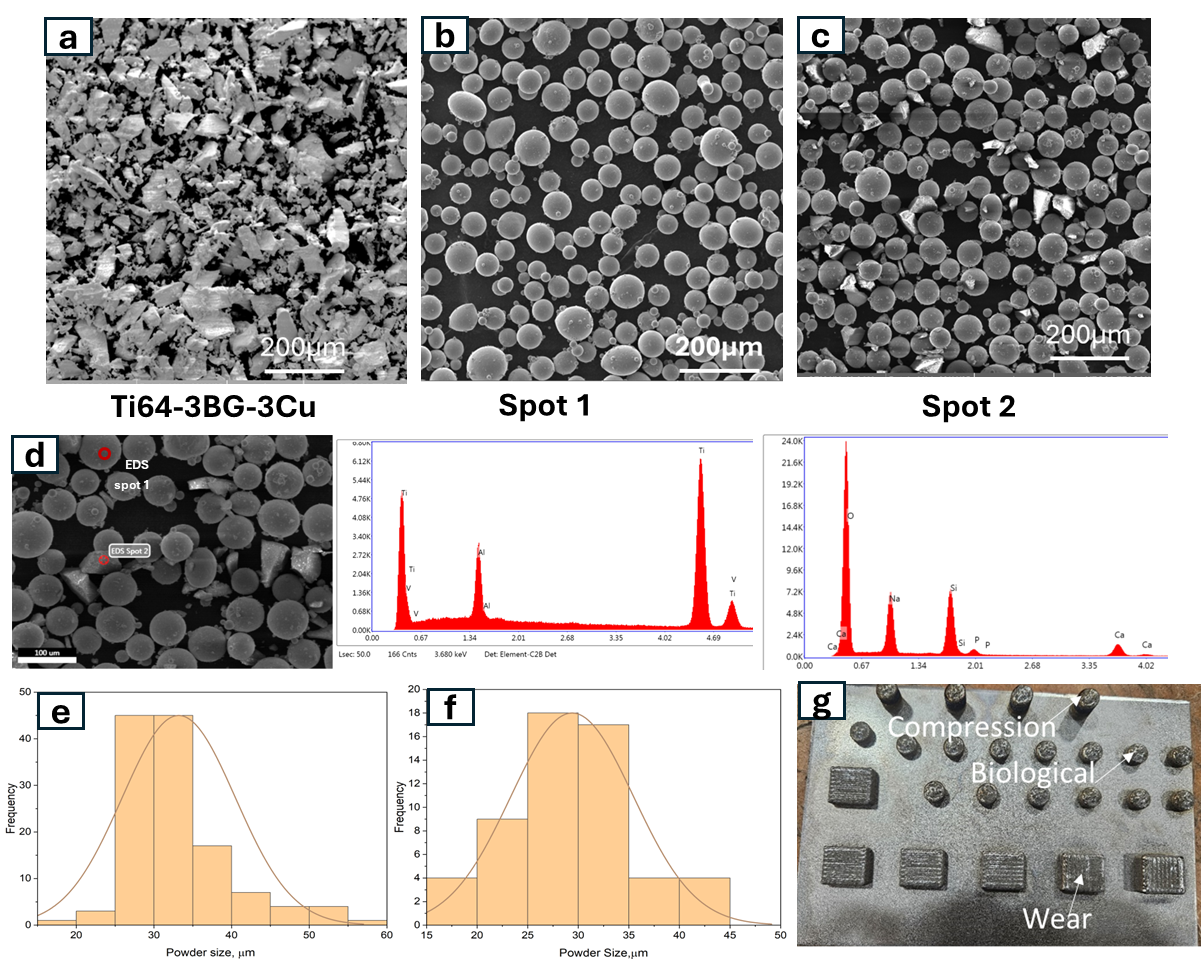


Figure 1: (a), (b), (c) show powder morphology of bioglass, Ti64, and Ti64-3BG-3Cu, respectively. (d) Is the micrograph of Ti64-3BG-3Cu for EDS analysis, the EDS spots 1 and 2 correspond to Ti64 and bioglass powder, as confirmed by EDS. Powder size distribution (e) Ti64, (f) Ti64-3BG-3Cu. Powder particles show unimodal distribution with a mean size of about 30 µm. (g) As printed, L-DED wear, compression, and compression testing samples on the CP-Ti base plate.

Nanoindentation was performed to determine the modulus of elasticity of all samples using Ti 950 TriboIndentor and shown in **Figure 2**. Before indentation, samples were ground and polished, and finally polished in Vibromet using 50 nm colloidal silica. Nanoindentation tests were performed using a Berkovich diamond indenter with a maximum load of 10 mN and a dwell time of 5 s, at a constant loading and unloading rate of 0.2 mN/s. Tests were performed in an 8 x 8 grid pattern with a spacing of 15 µm to minimize interaction effects and ensure an independent deformation zone. Hardness and elastic modulus were calculated using the Oliver-Pharr method, where reduced modulus was obtained from the unloading curve [1]. Actual modulus was calculated using the equation below.

$$\frac{1}{E_{r}}=\frac{1-{V_{s}}^{2}}{E_{S}}+\frac{1-{V_{i}}^{2}}{E_{i}}$$

E_r ,_ E_s_ , E_i_ is the reduced modulus, actual sample modulus and modulus of indenter respectively, whereas V_s ,_ V_i_ is the poisson’s ratio of sample and indenter respectively (E_i_.is 1141 GPa, V_i_ =0.07, V_s_ =0.34). A typical loading and unloading curves can be observed across all compositions. As shown in **Table 1** compositional variations did not change the modulus of elasticity, which is about 140 Gpa. The elastic modulus of Ti64 has been previously reported ti vary between 130-160 GPa [2], [3]. A clear trend can be seen on penetration depth, indicating improved hardness upon adding Bioglass and copper. These results are on-line with the Vickers test shown in figure 2 of main paper.

Table 1: Modulus and hardness of all composition measured using nanoindentation

| Compositions | Modulus (GPa) |
| --- | --- |
| Ti64 | 141 ± 17 |
| Ti64-1BG | 136 ± 5 |
| Ti64-3BG | 145 ± 11 |
| Ti64-3BG-3Cu | 143 ± 14 |


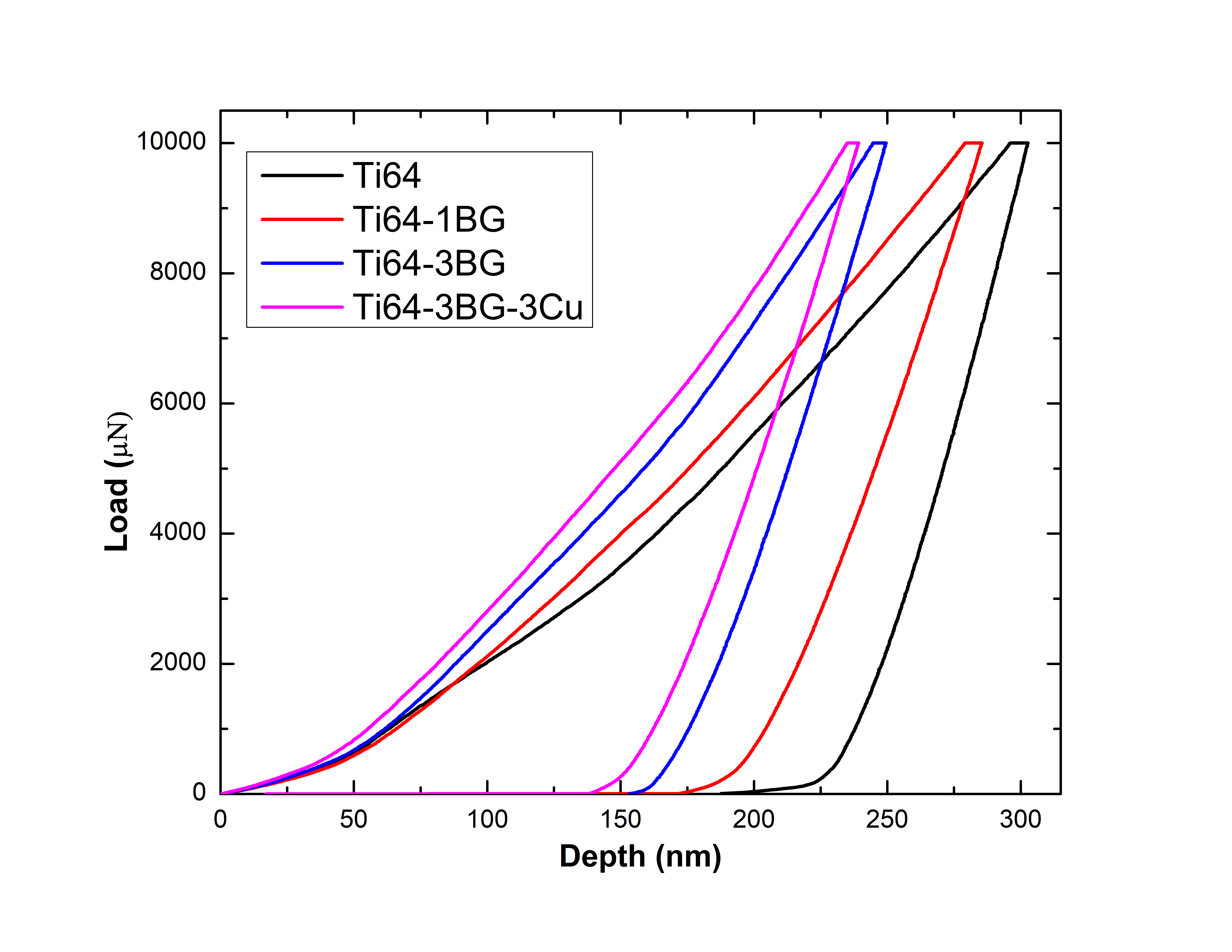


Figure 2 Representative nano indentations plot for Ti64, Ti64-1BG, Ti64-3BG, Ti64-3BG-3Cu samples.

`


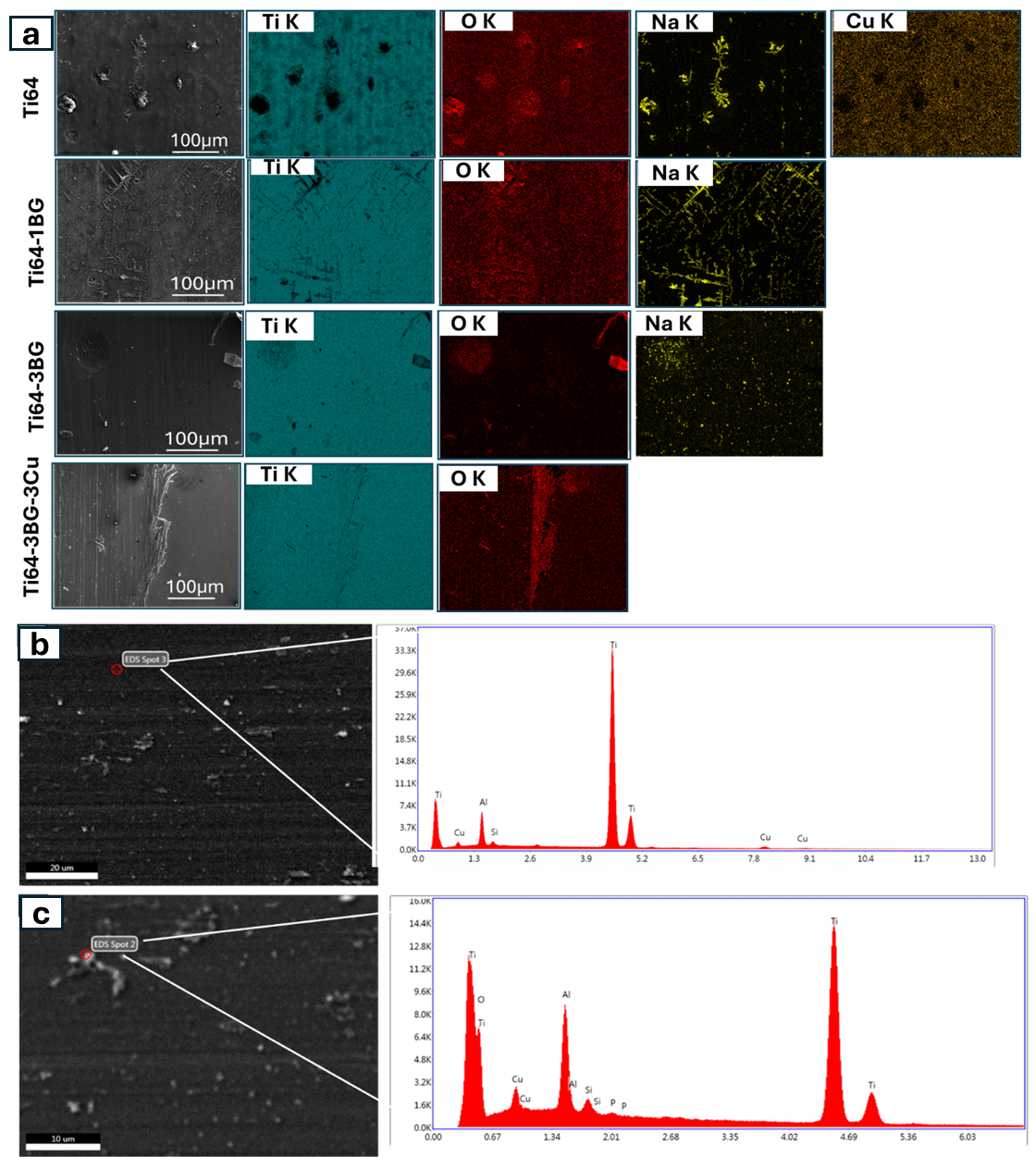


Figure 3 (a) EDS of wear track for all compositions for 5N load. Copper is uniformly distributed all over the sample. Bioglass particles are present along the wear track, as evident from the higher intensity of oxygen and sodium. Point EDS on the wear track of Ti64-3BG-3Cu. (a) Point EDS dark region. (b) Point EDS on white particles shows smeared Bioglass particles, as evident by the presence of silicon and phosphorus. Point EDS on the wear track of Ti64-3BG-3Cu. (b) Point EDS dark region. (c) Point EDS on white particles shows smeared Bioglass particles, as evident by the presence of silicon and phosphorus.


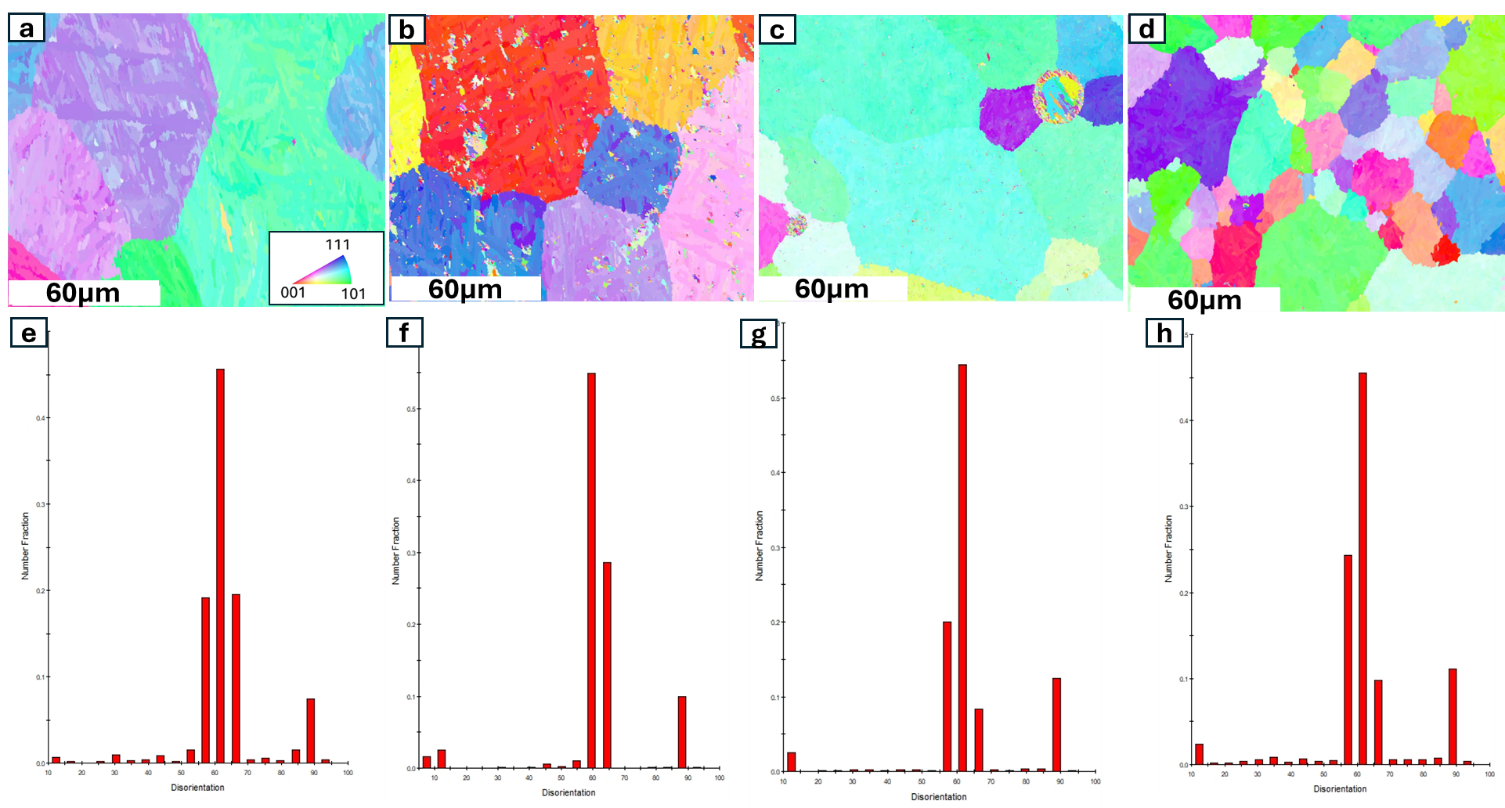


Figure 4: Micrographs showing prior Beta grain for (a) Ti64, (b) Ti64-1BG, (c) Ti64-3BG, (d) Ti64-3BG-3Cu. (e,f,g.h) shows disorientation plot for Ti64, Ti64-1BG, Ti64-3BG and Ti64-3BG-3Cu respectively.

X-ray fluorescence spectroscopy (XRF) was used to measure ionic release as described by the measurement technique in [4]. All composition samples and commercially pure copper samples with equal exposed surface area were immersed in Stimulated bodily fluid (SBF) at 37 °C in a shaker for 3 days to evaluate ion release. Before sampling, the ionic solution was thoroughly homogenized by vortexing, then digested in nitric acid at 80°C for 5 hours. Standard gallium solutions of known concentrations were added for calibration purposes. For analysis, 10 μL of each prepared solution was loaded onto servo-coated glass discs, and X-ray Fluorescence (XRF) spectroscopy was performed using molybdenum tubes operating at 50 kV. Blank SBF solution processed through identical preparation steps served as a reference to account for background signals from SBF components, nitric acid, and the servo-coated discs. The XRF measurements revealed no detectable release of titanium or copper ions from the Ti64-3BG-3Cu composites, as the ionic concentrations were comparable to those found in the baseline SBF solution over 3 days. However, with 14 and 28 days, Ti64-3BG-3Cu releases copper, which is about 2 and 5 ppm higher than the blank solution. In contrast, commercially pure copper samples exhibited significantly higher copper ion release rates. Ti64-3BG-3Cu copper ion concentration is less than 0.25% compared to that of commercially pure copper. As shown in **Figure 5,** the relative copper ion concentration release rate keeps decreasing compared to commercially pure copper. These XRF finding suggests initial antibacterial killing is through a contact killing mechanism, and with time, copper ion release also contributes to some extent.


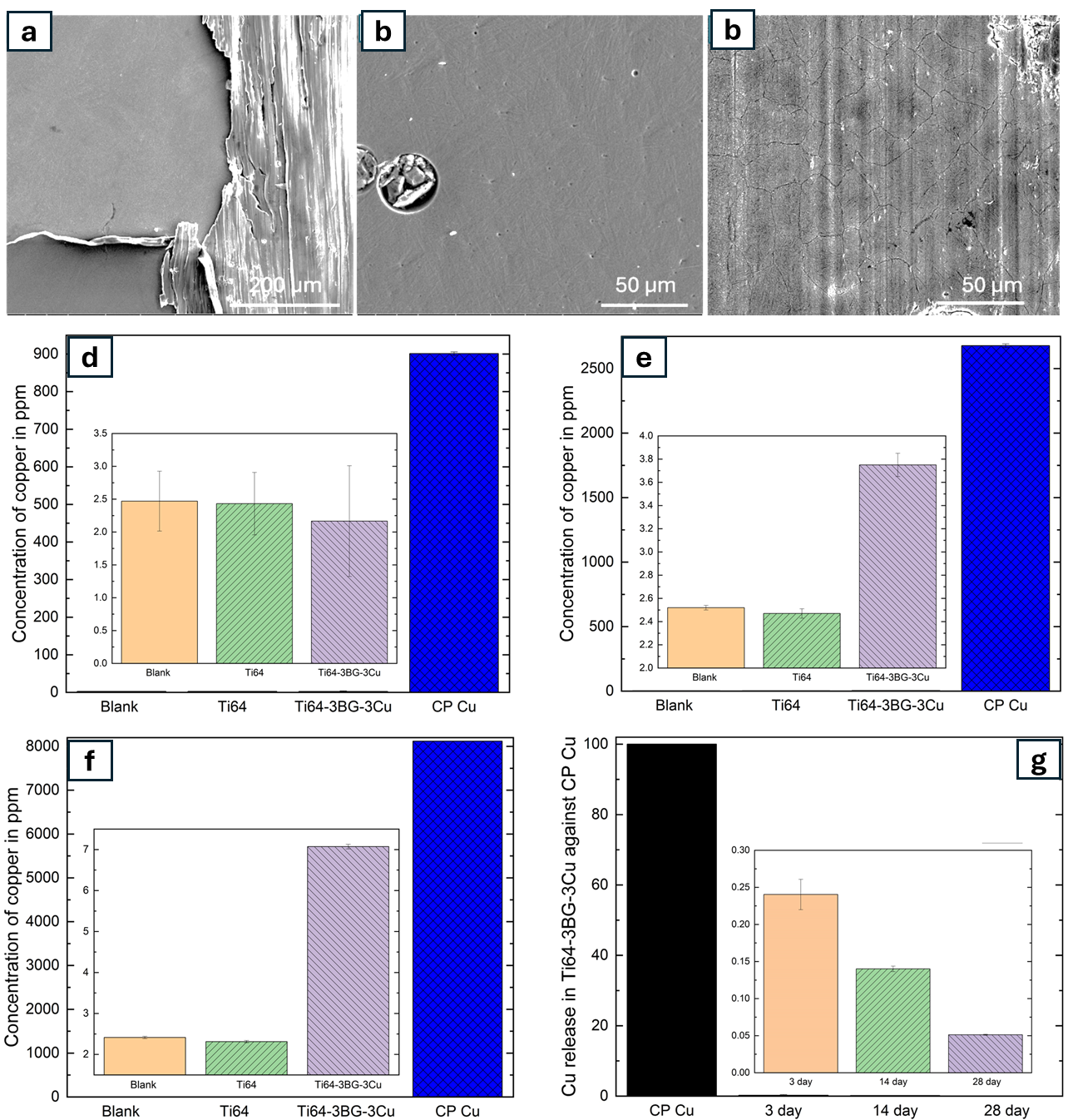


Figure 5: Microstructures of the wear track of 10N (a,b,c). (a) The non-wear track region of Ti64 shows acicular microstructures, whereas wear tracks show localized corrosion damage. (b, c) wear track morphology of Ti64-1BG and Ti64-3BG-3Cu. Ionic concentration of copper in blank, Ti64, Ti64-3BG-3Cu & commercially pure copper (d) 3 days, (e) 14 days, (f) 28 days. (g) shows relative copper release in Ti64-3Bg-3Cu compared to commercially pure copper.

**References**

[1] W. C. Oliver and G. M. Pharr, “An improved technique for determining hardness and elastic modulus using load and displacement sensing indentation experiments,” *Journal of Materials Research 1992 7:6*, vol. 7, no. 6, pp. 1564–1583, Jan. 2011, doi: 10.1557/JMR.1992.1564.

[2] D. Liović, M. Franulović, E. Kamenar, and D. Kozak, “Nano-Mechanical Behavior of Ti6Al4V Alloy Manufactured Using Laser Powder Bed Fusion,” *Materials*, vol. 16, no. 12, p. 4341, Jun. 2023, doi: 10.3390/MA16124341.

[3] C. Sánchez de Rojas Candela *et al.*, “A One-Step Novel Method to Fabricate Multigrade Ti6Al4V/TiN Composites Using Laser Powder Bed Fusion,” *Coatings 2024, Vol. 14, Page 90*, vol. 14, no. 1, p. 90, Jan. 2024, doi: 10.3390/COATINGS14010090.

[4] A. Bandyopadhyay, C. L. Orozco, L. Upadhayay, and A. Dash, “Hydroxyapatite-Reinforced, Infection-Resistant CoCrMo-3Cu for Load-Bearing Implants,” *ACS Appl Mater Interfaces*, Jul. 2025, doi: 10.1021/acsami.5c08994.
