# Supplementary figures and images for: Ti6Al4V‐Bioglass‐Copper Composites for Load‐Bearing Implants
Source: Adv Healthc Mater. 2026 Jan 26;15(13):e04606. doi: 10.1002/adhm.202504606 (PMC13058786; doi:10.1002/adhm.202504606)

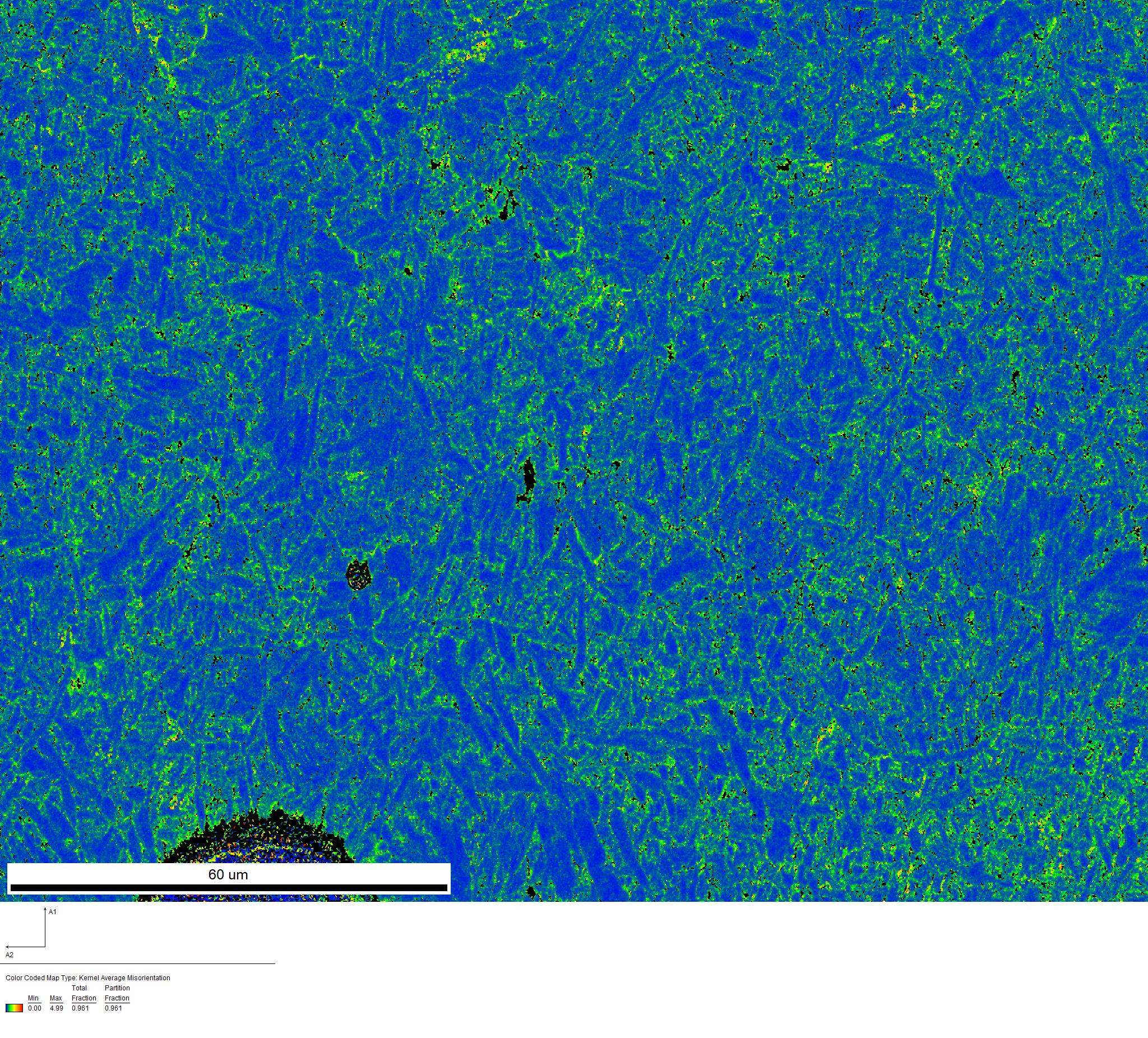

Supplement: Supplementary file 2 — Supporting File 2: adhm70775‐sup‐0002‐3cu KAM ‐ Fig 1h.jpg. [file ADHM-15-0-s001.jpg]

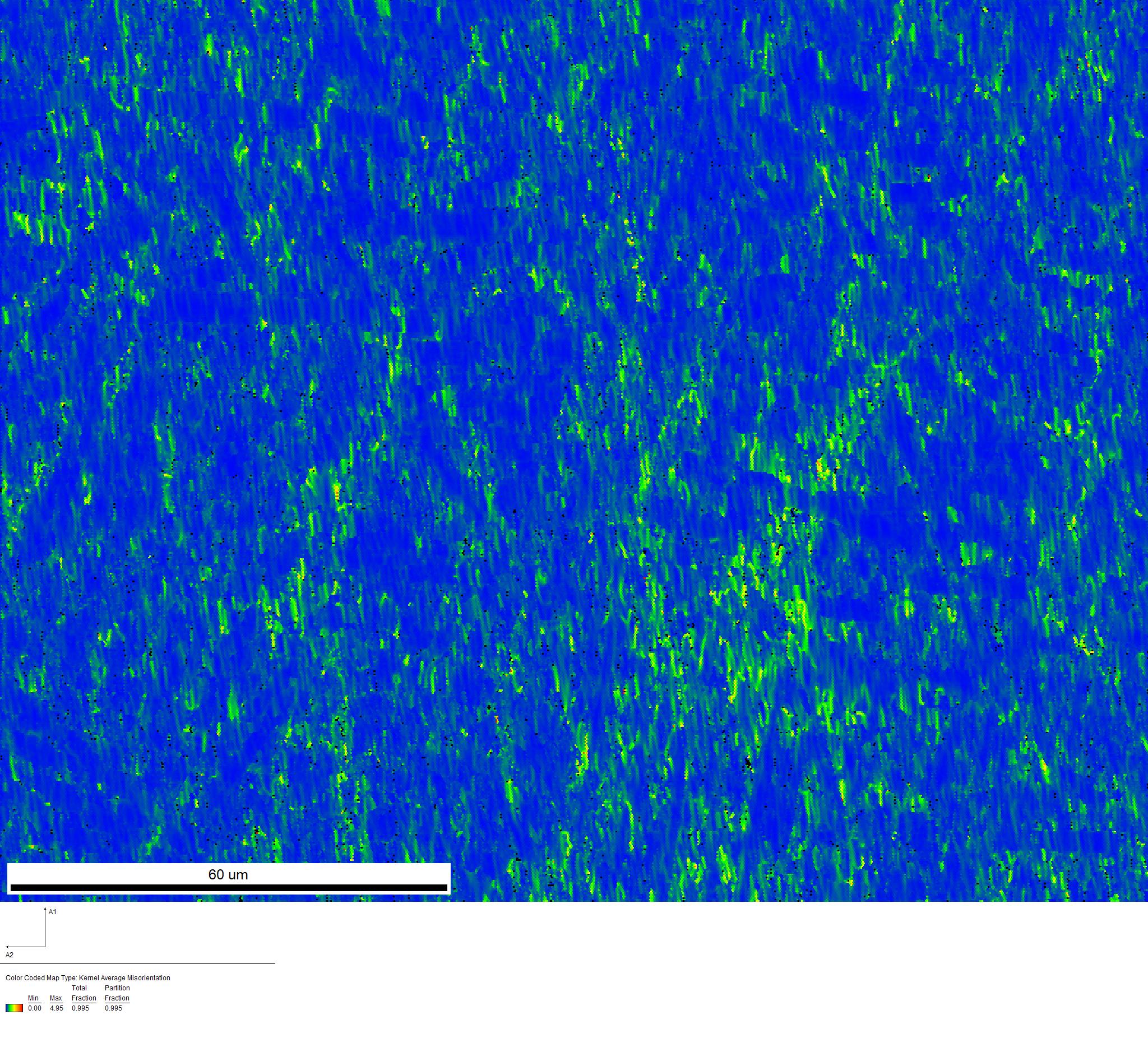

Supplement: Supplementary file 3 — Supporting File 3: adhm70775‐sup‐0003‐Ti64 KAM ‐ Fig 1e.jpg. [file ADHM-15-0-s002.jpg]
